# Advances in Flexible Thermoelectric Materials and Devices Fabricated by Magnetron Sputtering

**DOI:** 10.1002/smsc.202300061

**Published:** 2023-07-18

**Authors:** Boxuan Hu, Xiao-Lei Shi, Tianyi Cao, Meng Li, Wenyi Chen, Wei-Di Liu, Wanyu Lyu, Tuquabo Tesfamichael, Zhi-Gang Chen

**Affiliations:** ^1^ School of Chemistry and Physics Queensland University of Technology Brisbane Queensland 4001 Australia; ^2^ School of Mechanical and Mining Engineering The University of Queensland Brisbane Queensland 4072 Australia; ^3^ Australian Institute for Bioengineering and Nanotechnology The University of Queensland Brisbane Queensland 4072 Australia; ^4^ School of Mechanical, Medical and Process Engineering Queensland University of Technology Brisbane Queensland 4001 Australia

**Keywords:** inorganics, magnetron sputtering, thermoelectrics, thin films

## Abstract

Due to the direct conversion between thermal and electrical energy, thermoelectric materials and their devices exhibit great potential for power generation and refrigeration. With the rapid development of personal wearable electronics, the design of flexible inorganic thermoelectric materials and devices receives increasing attention. As one of the most mature thin‐film fabrication techniques, magnetron sputtering plays a key role in the fabrication of inorganic thermoelectric thin films and devices, but its progress is still not timely and comprehensively reviewed. Herein, recent advances in magnetron sputtering‐fabricated thermoelectric materials and devices are studied, including their thermoelectric properties, mechanical properties, and device design routes. The differences in the properties of thermoelectric materials under different sputtering conditions, as well as their underlying mechanisms, are carefully discussed. In the end, it is pointed out the challenges and future directions for magnetron sputtering‐prepared inorganic thermoelectric thin‐film materials and devices for practical applications. This review can serve as a useful reference to guide the design of inorganic thermoelectric materials and devices prepared by magnetron‐sputtering‐based deposition techniques.

## Graphical abstract

Magnetron sputtering is an important technique for thermoelectric thin‐film fabrication. A critical review on concluding the influence of sputtering pressure, substrate type, substrate temperature, annealing, hybridization, doping, etc. on sputtered thermoelectric thin films is reported, understanding the thermoelectric and mechanical performance. This review may help guide the design of magnetron‐sputtered thermoelectric materials and devices.

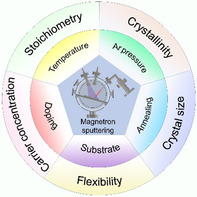

## 1 Introduction

Traditional fossil energy sources induce the giant greenhouse effect and air pollution, leading to considerable attention for exploring ecofriendly energy conversion techniques.^[^ ^1^, ^2^, ^3^, ^4^, ^5^, ^6^, ^7^, ^8^ ^]^ Thermoelectrics, enabling direct conversion between heat and electricity, have become potential candidates for tackling the above problems. Compared with other zero‐emission energy conversion techniques, thermoelectric devices (TEDs), composed of thermoelectric materials, exhibit unique characteristics, including fast response, high stability, long life, and low maintenance.^[^ ^2^, ^9^, ^10^, ^11^, ^12^, ^13^ ^]^ TEDs rely on the Seebeck effect, Peltier effect, and Thomson effect during the operation. Seebeck effect allows a thermoelectric material to generate electricity using temperature difference (Δ*T*).^[^ ^14^, ^15^, ^16^ ^]^ The Peltier effect refers to the transfer of heat from one side of a thermoelectric material to the other when driven by an electrical force. The Thomson effect refers to the fact that a thermoelectric material can absorb or release heat from itself at an arbitrary Δ*T*, which is determined by the direction of the current and the material.^[^ ^17^, ^18^, ^19^, ^20^ ^]^ Generally, thermoelectric generators (TEGs) rely on the Seebeck effect and work under a certain Δ*T*, and thermoelectric coolers (TECs) rely on the Peltier effect driven by direct current (DC).^[^ ^2^, ^21^, ^22^, ^23^, ^24^, ^25^, ^26^ ^]^ Within these devices, the dimensionless figure of merit *ZT* is one of the most important parameters to determine the thermoelectric potential of thermoelectric materials, expressed as^[^ ^2^ ^]^

(1) $$ZT=\frac{S^{2}\sigma T}{\kappa }\;$$

where *S* is the Seebeck coefficient, *κ* is the thermal conductivity, *σ* is the electrical conductivity, and *T* is the absolute temperature. Higher *ZT* values always contribute to high performance in both TEGs and TECs.^[^ ^2^, ^27^, ^28^, ^29^, ^30^ ^]^ Therefore, it is of significance to develop high‐*ZT* thermoelectric materials for improving the overall performance of TEDs. TEDs have been used in a wide range. For example, TEDs, composed of Bi_2_Te_3_ and PbTe materials, have been employed in space exploration and industrial waste heat recovery since the 1950s.^[^ ^31^, ^32^, ^33^ ^]^ Recently, broadened applications have been realized for innovatively designed TEDs such as flexible and miniature TEDs to meet the demands for portable and wearable electronics, the Internet of Things (IoT), chip thermal management, and personal health tracking.^[^ ^34^, ^35^, ^36^ ^]^ Although the current TEDs still show limited performance,^[^ ^37^, ^38^, ^39^, ^40^ ^]^ they are still promising and expected to be useful for commercialization in the future after further understanding the fundamentals and in turn, improving thermoelectric properties.^[^ ^9^, ^41^, ^42^, ^43^, ^44^ ^]^

Currently, three different configurations, including 3D bulk‐based TEDs, 2D thin‐film‐based TEDs, and 1D fiber‐based TEDs,^[^ ^43^ ^]^ are widely used. Among these configurations, 3D bulk‐based TEDs exhibit good performance and were initially commercialized,^[^ ^34^, ^45^ ^]^ However, the rigidity of the configuration makes these TEDs difficult in applying to flexible and wearable applications.^[^ ^46^ ^]^ Oppositely, thin‐film‐ and fiber‐based TEDs exhibit better flexibility.^[^ ^43^ ^]^ However, limited by current fabrication techniques and the lack of fundamental understanding, fiber‐based TEDs are still difficult to be applied due to their low stability and insufficient output performance.^[^ ^2^ ^]^ Therefore, thin‐film‐based TEDs are good candidates for realizing flexible and portable power generation and refrigeration by balancing performance and flexibility. The performance of thin‐film‐based TEDs is highly dependent on their thicknesses,^[^ ^47^ ^]^ crystallographic orientations,^[^ ^48^ ^]^ and densities.^[^ ^49^ ^]^ These factors are considerably determined by the thin‐film preparation methods. Generally, thin films can be made by chemical^[^ ^50^ ^]^ and physical methods.^[^ ^51^ ^]^ However, historically, thermoelectric thin films fabricated by chemical methods usually exhibit worse performance than that fabricated from physical routes due to their relatively unstable structures.^[^ ^52^ ^]^ Therefore, increasing attention is paid to high‐performing thermoelectric thin films and devices prepared by physical methods, which are better choices for assembling wearable/portable electronics for both power generation and localized cooling.

**Figure 1:**
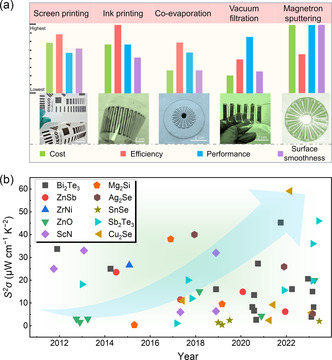
Progress on different thermoelectric materials and devices. a) Comparison of features (cost, efficiency, performance, surface smoothness) for different thin‐film fabrication routes including screen printing, ink printing, evaporating, vacuum filtration, and magnetron sputtering (The cost is the equipment cost, the efficiency is the sputtering rate, the performance is the power factor of the prepared film, and the surface smoothness is the smoothness of the film surface). The corresponding photos for the as‐fabricated thin films and devices are included as insets, including, from left to right, silkscreen Ag_2_Se‐based films prepared by screen printing, reproduced with permission.^[^ ^53^ ^]^ Copyright 2020, American Chemical Society, nitrogen‐doped graphene films prepared by ink printing, reproduced with permission.^[^ ^54^ ^]^ Copyright 2016, Elsevier, Ag_2_Se films prepared by coevaporation, reproduced with permission.^[^ ^55^ ^]^ Copyright 2022, Elsevier, Ag_2_Se films prepared by vacuum filtration, reproduced with permission.^[^ ^56^ ^]^ Copyright 2020, Royal Society of Chemistry, and Bi_2_Te_3_ film prepared by magnetron sputtering, reproduced with permission.^[^ ^8^ ^]^ Copyright 2022, Springer Nature. b) Peak power factor *S* ^2^ *σ* of inorganic thin films prepared by magnetron sputtering techniques reported in recent years including Bi_2_Te_3_,^[^ ^8^, ^51^, ^61^, ^62^, ^63^, ^64^, ^65^, ^66^, ^67^, ^68^, ^69^, ^170^, ^171^, ^172^ ^]^ ZnSb,^[^ ^70^, ^71^, ^72^, ^73^ ^]^ Zr–Ni–Sn,^[^ ^74^ ^]^ ZnO,^[^ ^75^, ^76^, ^77^, ^78^, ^79^, ^173^ ^]^ ScN,^[^ ^80^, ^81^, ^82^, ^83^, ^84^ ^]^ Cu_2_Se,^[^ ^85^, ^86^, ^87^, ^88^, ^89^, ^174^ ^]^ Sb_2_Te_3_,^[^ ^90^, ^91^, ^92^, ^93^, ^94^, ^175^, ^176^ ^]^ Ag_2_Se,^[^ ^95^, ^96^, ^177^ ^]^ SnSe,^[^ ^97^, ^98^, ^99^, ^178^ ^]^ MgSi^[^ ^100^, ^101^, ^102^ ^]^‐based thin films.

The common physical fabrication routes, include screen printing,^[^ ^53^ ^]^ ink printing,^[^ ^54^ ^]^ coevaporation,^[^ ^55^ ^]^ vacuum filtration,^[^ ^56^ ^]^ and magnetron sputtering,^[^ ^8^, ^57^, ^58^ ^]^ are used to fabricate thin‐film‐based thermoelectric materials. **Figure** **1**a compares the characteristics of different methods for fabricating thermoelectric thin films from the aspects, including cost, efficiency, thermoelectric performance, and surface smoothness. Compared to other deposition processes, magnetron sputtering allows the sputtering of uniform inorganic thin films with high performance on variable substrates with precise thickness control and good adhesion.^[^ ^59^, ^60^ ^]^ Till now, there have been various material systems fabricated by magnetron sputtering techniques, including Bi_2_Te_3_,^[^ ^8^, ^51^, ^61^, ^62^, ^63^, ^64^, ^65^, ^66^, ^67^, ^68^, ^69^ ^]^ ZnSb,^[^ ^70^, ^71^, ^72^, ^73^ ^]^ Zr–Ni–Sn,^[^ ^74^ ^]^ ZnO,^[^ ^75^, ^76^, ^77^, ^78^, ^79^ ^]^ ScN,^[^ ^80^, ^81^, ^82^, ^83^, ^84^ ^]^ Cu_2_Se,^[^ ^85^, ^86^, ^87^, ^88^, ^89^ ^]^ Sb_2_Te_3_,^[^ ^90^, ^91^, ^92^, ^93^, ^94^ ^]^ Ag_2_Se,^[^ ^95^, ^96^ ^]^ SnSe,^[^ ^97^, ^98^, ^99^ ^]^ and MgSi^[^ ^100^, ^101^, ^102^ ^]^‐based thin films in recent years. For comparison, the peak power factors, *S* ^2^ *σ*, of these materials are shown in Figure 1b,^[^ ^8^, ^51^, ^61^, ^62^, ^63^, ^64^, ^65^, ^66^, ^67^, ^68^, ^69^, ^70^, ^71^, ^72^, ^73^, ^74^, ^75^, ^76^, ^77^, ^78^, ^79^, ^80^, ^81^, ^82^, ^83^, ^84^, ^85^, ^86^, ^87^, ^88^, ^89^, ^90^, ^91^, ^92^, ^93^, ^94^, ^95^, ^96^, ^97^, ^98^, ^99^, ^100^, ^101^, ^102^ ^]^ from which a high *S* ^2^ *σ* of ≈60 μW cm^−1^ K^−2^ has been achieved in Cu_2_Se thin films fabricated by this method,^[^ ^88^ ^]^ which is an outstanding value. Considering the rapid development of magnetron sputtering‐fabricated thermoelectric thin films in these years, a comprehensive review is required to timely summarize advances in thermoelectric thin films and devices made by magnetron sputtering techniques.

In this review, we first focus on the principles of magnetron sputtering and point out its advantages and disadvantages compared to other thin‐film‐fabrication techniques. After that, we carefully discuss differences in thermoelectric properties and their flexibility and compare properties and features between magnetron‐sputtering‐based thin films and those fabricated by other routes. In the end, we point out the challenges and outlook for magnetron sputtering‐fabricated thermoelectric thin films and devices for future applications.

## 2 Principle of Magnetron Sputtering

### 2.1 Basic Parameters

In classical thermodynamics, the Carnot effect represents the maximum efficiency of a reversible heat machine. For TEDs, it is determined by Δ*T* between the two sides of the device and the properties of the thermoelectric materials. The Carnot efficiency *ϕ*_c_ can be expressed as

(2) $$\phi c=\frac{T_{H}-T_{C}}{T_{H}}$$

where *T*_C_ and *T*_H_ refer to the cold‐side and hot‐side temperatures. The maximum thermoelectric conversion efficiency *η*_max_ of the TED is obtained by combining the Carnot efficiency with the *ZT* expressed as

(3) $$\eta_{\text{max}}=\left(\frac{T_{H}-T_{C}}{T_{H}}\right)\cdot \left(\frac{\sqrt{1+ZT}-1}{\sqrt{1+ZT}+\frac{T_{C}}{T_{H}}}\right)$$

From Formula (3), it can be seen that higher *ZT*s contribute to higher *η*_max_ (here *ZT*s refer to the *ZT* values of multiple materials). Till now, many strategies have been reported to effectively improve the *ZT*s of inorganic thermoelectric materials including both bulk materials and thin films,^[^ ^2^, ^9^, ^10^, ^11^, ^12^, ^39^, ^43^, ^45^, ^66^, ^103^ ^]^ which will not be discussed in detail. Generally, these strategies are suitable for designing inorganic thermoelectric thin films fabricated by magnetron sputtering routes.

### 2.2 Fundamentals

Magnetron sputtering is a widely used method to prepare inorganic thermoelectric films.^[^ ^67^, ^104^, ^105^, ^106^, ^107^, ^108^, ^109^ ^]^ This method produces a cascade of atomic collisions by bombardment with high‐energy ions, causing target atoms to escape from the target to the surface of the substrate.^[^ ^110^ ^]^ During sputtering, argon is often used as the processing gas due to low cost and high sputter yield.^[^ ^40^, ^57^, ^59^, ^104^, ^110^ ^]^ From sputtering, films of high quality and uniformity, with high adhesion to the substrate, can be achieved using tunable sputtering parameters such as pressure, power and temperature. Reactive magnetron sputtering can be carried out at room temperature as a method to create continuous inorganic films on flexible substrates, realizing certain flexibility in the as‐deposited thin films.^[^ ^57^, ^59^, ^104^, ^110^ ^]^ However, if applying a small amount of reactive gas such as nitrogen or oxygen into the chamber, the reactive gas can react with the target and produce a new compound film on the surface of the substrate.^[^ ^110^ ^]^ By controlling the amount of reactive gas, doping can be regulated, or a fully reacted compound can be produced.^[^ ^110^ ^]^ Magnetron sputtering can produce thicker, denser, and homogeneous inorganic thermoelectric films with strong bonding to the substrate and allows the deposition of films at room temperature.^[^ ^104^ ^]^ In addition, during the synthesis, it can precisely regulate the sputtering pressure and annealing temperature to synthesize inorganic thermoelectric films with different properties and structures.^[^ ^57^, ^104^ ^]^ Therefore, thin films made by magnetron sputtering exhibit a unique performance than those made by other methods.^[^ ^110^ ^]^ Although magnetron sputtering has some limitations such as expensive equipment and low sputtering rates,^[^ ^59^ ^]^ it is still one of the most suitable methods for the preparation of large‐sized inorganic thermoelectric films with high performance.

### 2.3 Sputtering Conditions

**Figure 2:**
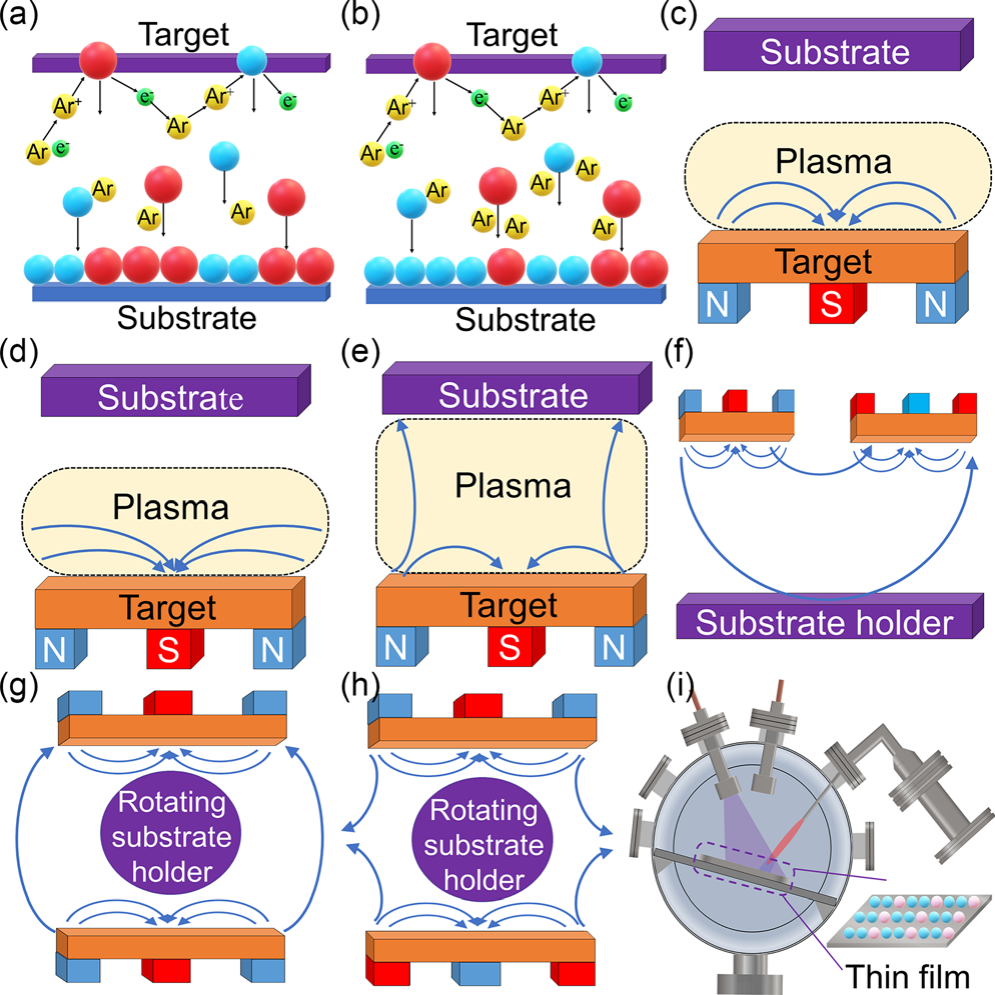
Schematic models of different magnetron sputtering. a,b) Effects of low and high Ar pressure on magnetron sputtering.^[^ ^111^ ^]^ c) Schematic diagram of plasma confinement for balanced magnetrons. Schematic diagram of plasma confinement for unbalanced magnetrons: d) Type‐1 and e) Type‐2. f–h) Three double unbalanced magnetron configurations including planar closed domain configuration, vertical closed domain configuration, and mirror image configuration, respectively.^[^ ^60^ ^]^ i) Schematic diagram of the PHRMS.^[^ ^96^ ^]^.

The features and performance of thin films are considerably affected by the conditions of magnetron sputtering. For example, the different pressures of argon in the chamber can impact the compositions of the as‐synthesized films,^[^ ^60^, ^111^ ^]^ as shown in **Figure** **2**a,b. Under low argon pressure, different sizes of atoms can be deposited on the substrate; however, this situation may result in a disproportion of atoms in the films and affect the performance of the as‐fabricated thin films.^[^ ^60^ ^]^ On the contrary, high argon pressure may inhibit the deposition of larger‐sized atoms, leading to relatively controllable compositions.^[^ ^111^ ^]^ However, if the argon pressure is excessive, the sputtering of all atoms may be severely inhibited. Therefore, a careful selection of argon pressure is important for different material systems.^[^ ^111^ ^]^

The magnetic field can influence the performance and features of thin films. Figure 2c shows a schematic diagram of plasma confinement for balanced magnetrons. As shown, an early magnetron sputtering machine can only maintain ≈60 mm magnetic field.^[^ ^60^ ^]^ Such a low distance may cause the substrate simultaneously to be bombarded by energetic plasma and target atoms, resulting in degraded thin films.^[^ ^60^ ^]^ Therefore, an unbalanced magnetron technique can be employed for tackling the problem.^[^ ^60^ ^]^ This type of magnetron sputtering can be further classified as Type‐1 and Type‐2,^[^ ^60^ ^]^ as illustrated in Figures 2d,e. Type‐1 magnetron sputtering reduces the plasma density in the chamber, which prevents the thin film damaged by plasma.^[^ ^60^ ^]^ Type‐2 magnetron sputtering enlarges the scale of the magnetic field, which leads to the target atoms being sputtered on the substrate beyond 60 mm and in turn, reducing the influence of plasma.^[^ ^60^, ^112^, ^113^ ^]^

To more rapidly prepare uniform thin films, magnetron sputtering equipment with multiple magnetron systems can be used.^[^ ^60^ ^]^ Closed loop is suitable to achieve this goal and can be divided into side‐view and top‐side systems,^[^ ^60^ ^]^ as illustrated in Figures 2f,g. In a closed‐field configuration, there is a relatively high concentration of sputtered target atoms in the chamber, which can accelerate the sputtering efficiency. The closed‐field configuration can effectively prevent the target atom from attaching to the chamber walls, which prevents the waste of targets. With increasing the number of magnetron tubes, the sputtering speed can be tuned. However, if the magnetron sputtering device is designed as a mirrored‐field configuration, the target atoms may be sputtered on the chamber wall, leading to low sputtering efficiencies and target wastes,^[^ ^60^ ^]^ as shown in Figure 2h.

Magnetron sputtering has rapidly developed over the last few decades.^[^ ^60^ ^]^ An important new technology for magnetron sputtering, called pulsed hybrid reactive magnetron sputtering (PHRMS), has been developed.^[^ ^85^, ^96^ ^]^ As illustrated in Figure 2i, PHRMS does not require high‐temperature postannealing to obtain good thermoelectric properties, and the films can be sputtered on a polymer‐based substrate.^[^ ^85^ ^]^ This technique can avoid chamber poisoning that tends to occur with conventional magnetron sputtering and ensure the integrity of films during growth.^[^ ^85^, ^96^ ^]^ Such a technique is used to prepare selenide films,^[^ ^85^, ^96^ ^]^ where the metal target is first deposited for initial growth, and then atoms from the selenium vapor are introduced into the chamber by periodic pulses.^[^ ^85^, ^96^ ^]^ This process allows finely controlled selenium content in the synthesized film and accelerates the growth rate,^[^ ^96^ ^]^ thus optimizing the binding kinetics of the anions and cations on the surface of films.^[^ ^85^ ^]^

### 2.4 Comparison with Other Thin‐Film Fabrication Techniques

Apart from the magnetron sputtering method, there are other methods for fabricating thermoelectric thin films, such as screen printing, pulsed laser deposition (PLD), and thermal coevaporation. For screen printing, the cost of this method is lower than magnetron sputtering and allows to quick fabrication of thermoelectric thin films.^[^ ^114^ ^]^ However, this method is difficult to achieve complete reactions between precursors. For example, Bi_2_Te_3_ thin film fabricated by this method usually had a rough surface, and the components of this thin film were mainly Bi and Te monomers.^[^ ^114^ ^]^ In this situation, annealing is a compulsory strategy for supplying energy to promote the reaction of Bi and Te monomers. The as‐fabricated thin film showed a *S* ^2^ *σ* of 21 μW cm^−1^ K^−2^ and a *ZT* of 0.61.^[^ ^114^ ^]^ Compared with the thin film made by magnetron sputtering, the screen‐printed thin film exhibited a better *S* ^2^ *σ*, but this thin film had a relatively high *κ*, leading to a lower *ZT*. In addition, the annealing treatment temperature was usually 773 K, which is not suitable for employing organic‐based substrates, and the as‐fabricated thin film may have relatively low ductility and flexibility.^[^ ^114^ ^]^ As for the PLD method, the thin film made of this method had good stoichiometry with a smooth and dense surface structure. However, the PLD method requires high costs and relies heavily on the control of the substrate temperature, which may result in unstable thermoelectric properties in the as‐fabricated thin film.^[^ ^115^ ^]^ Besides, the performance of such thin films is relatively lower than that fabricated by magnetron sputtering.^[^ ^115^ ^]^ Thermal coevaporation is a common technique for fabricating thermoelectric thin films, which allows the as‐fabricated thin films to be uniform and dense with a high *S* ^2^ *σ* of 25.7 μW cm^−1^ K^−2^ for room temperature.^[^ ^116^ ^]^ However, compared with magnetron sputtering, this technique has challenges in substrate selection and component control.^[^ ^104^ ^]^ Therefore, magnetron sputtering is a suitable technique for fabricating thermoelectric materials with dense and stable structures and in turn good thermoelectric properties.

## 3 Strategies for Magnetron‐Sputtering‐Fabricated Inorganic Thermoelectric Films

### 3.1 Tuning Sputtering Power

Sputtering power is an important parameter that controls the microstructure and hence regulates the properties of thermoelectric thin films. It has been reported that adjusting the sputtering power can affect the film *σ* and *S*.^[^ ^117^ ^]^ A cosputtering method was used to prepare Zn_4_Sb_3_ thin films, and this process was performed by varying the sputtering power and sputtering time of the films, and the measurement of their *σ* can be found that the *σ* of the films increases with the increase of the film thickness when the sputtering power on both targets is kept constant.^[^ ^117^ ^]^ When the film thickness increases beyond a certain value, *σ* remains constant. However, when the film has the same thickness, the *σ* decreases as the sputtering power increases and the deposition time decreases, and the samples with lower *σ* have larger *S*.^[^ ^117^ ^]^ The presence of dispersed Zn‐rich particles in the films was observed by a scanning electron microscope (SEM), and the particle size increases with increasing film thickness.^[^ ^117^ ^]^ The analysis by energy‐dispersive spectroscopy (EDS) showed that the composition of the films prepared at different powers did not change.^[^ ^117^ ^]^ It was eventually found that the films with large thickness and coarse Zn‐rich particles in the microstructure had low *σ* high *S*. The films with moderate thickness and dispersed fine Zn‐rich particles showed the highest *S* ^2^ *σ*, and the 349 nm‐thick Zn_4_Sb_3_ thin film showed 12 μW cm^−1^ K^−2^ of *S* ^2^ *σ* and 1.2 of *ZT* at ≈460 K.^[^ ^117^ ^]^

### 3.2 Tuning Sputtering Pressures

**Figure 3:**
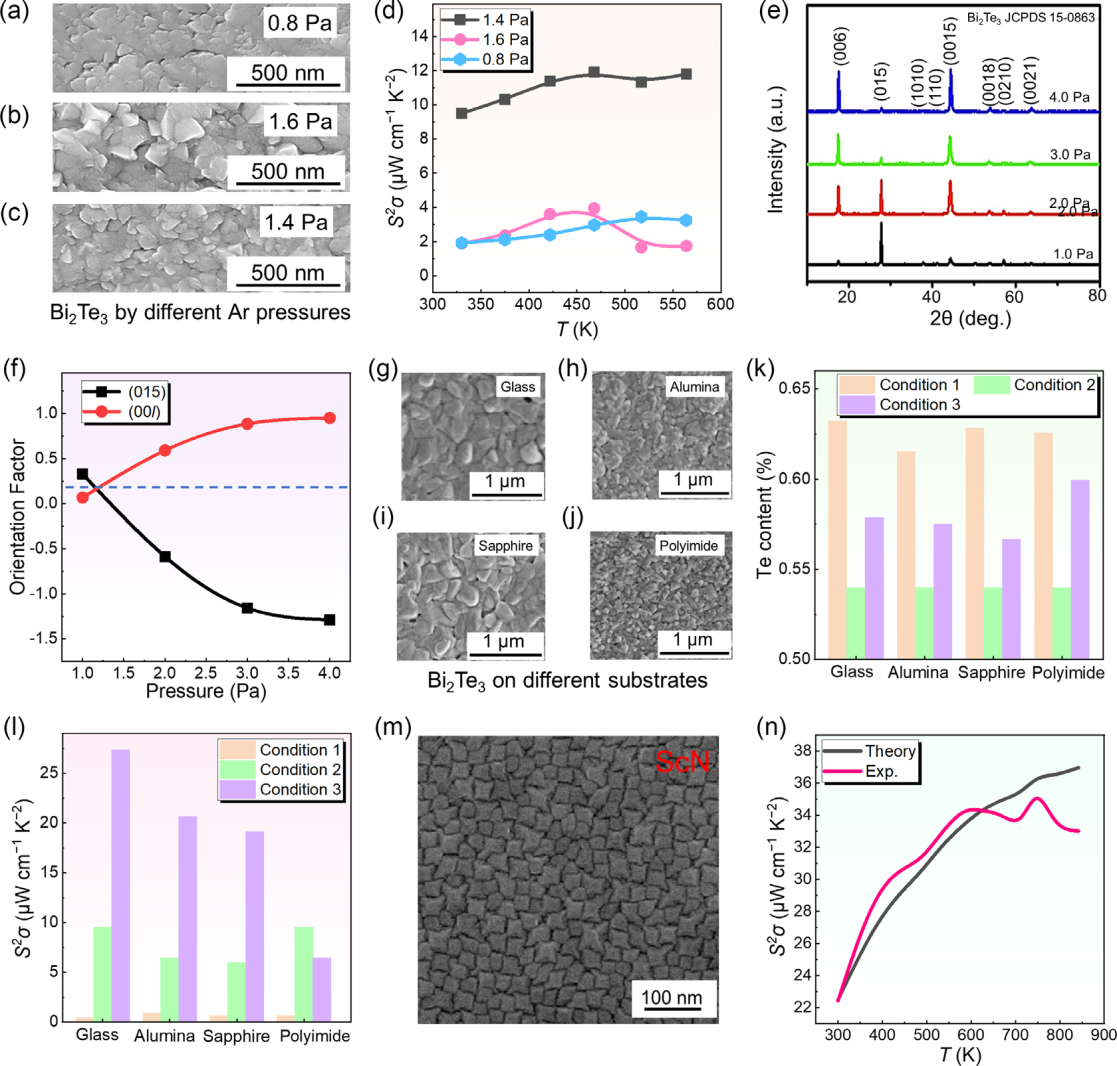
Effect of sputtering pressure and substrate selection on magnetron‐sputtered films. a–c) SEM images of Bi_2_Te_3_ films deposited under three different Ar pressures (0.8, 1.6, and 1.4). d) *S* ^2^ *σ* of the three films as functions of temperature. a–c) Reproduced with permission.^[^ ^111^ ^]^ Copyright 2017, Springer Nature. e) XRD patterns of Bi_2_Te_3_ films prepared under different sputtering pressures. f) Orientation factors of (015) and (00*l*) of Bi_2_Te_3_ films deposited under different sputtering pressures. e,f) Reproduced with permission.^[^ ^121^ ^]^ Copyright 2019, Elsevier. g–j) SEM images of Bi_2_Te_3_ films deposited on four different substrates including glass, alumina, sapphire, and polyimide (PI). k) Te contents and l) *S* ^2^ *σ* of the Bi_2_Te_3_ films prepared under different conditions on four different substrates. g–l) Reproduced with permission.^[^ ^61^ ^]^ Copyright 2020, Elsevier. m) SEM image of a ScN film. n) Theoretical and experimental *S* ^2^ *σ* of ScN films as functions of temperature. m,n) Reproduced with permission.^[^ ^80^ ^]^ Copyright 2013, AIP.

Sputtering pressure is an important parameter that controls the components and in turn the properties of thermoelectric thin films.^[^ ^111^ ^]^ It was reported that tuning the sputtering pressure can influence the composition ratio of Bi_2_Te_3_.^[^ ^9^, ^118^ ^]^ **Figures** **3**a–c shows SEM images of the Bi_2_Te_3_ thin films sputtered at 0.8, 1.6, and 1.4 Pa.^[^ ^111^ ^]^ Under an argon pressure of 0.8 Pa, the thin film surface was relatively smooth. When the sputtering pressure was increased, the film surface became rougher, which could influence the atomic agglomerations and surface diffusions.^[^ ^111^ ^]^ The thin films prepared under different argon pressures had different Te contents, which were measured as 49%, 54%, and 57%.^[^ ^111^ ^]^ According to the reported work, 0.8 Pa argon pressure was not suitable for sputtering Bi_2_Te_3_ thin film. Owing to the higher mass of the Bi atom than that of the Te atom, 0.8 Pa argon pressure was difficult to provide sputtering energy to make the Bi atom being sputtered on the substrate, resulting in a high Te content in the film.^[^ ^111^, ^119^, ^120^ ^]^ However, when the argon pressure was increased to 1.6 Pa, the high argon atoms in the chamber could simultaneously hinder the sputtering of Bi and Te elements, leading to rough thin films and wasting target materials.^[^ ^111^ ^]^ In terms of the output performance (Figure 3d), the highest *S* ^*2*^ *σ* of was 12 μW cm^−1^ K^−2^ at room temperature. However, the upper limit for Te content was 57%, which was still tricky to reach 60%.^[^ ^111^ ^]^

In addition to the composition ratio, the sputtering pressure affects the grain orientations and grain sizes of the thermoelectric thin films. It was reported that the orientation of Bi_2_Te_3_ grains gradually changed with increasing the sputtering pressure.^[^ ^121^ ^]^ Figure 3e shows the X‐ray diffraction (XRD) patterns of Bi_2_Te_3_ films prepared under different sputtering pressures.^[^ ^121^ ^]^ As can be seen, the thin film fabricated under 1.0 Pa exhibited a preferred orientation of (015). However, with increasing sputtering pressure, the (015) orientations were changed into (00*l*) orientations.^[^ ^121^ ^]^ In addition, the calculation result of the orientation factor showed that the orientation factor of (015) decreased from 0.32 to −1.28 and the (00*l*) orientation factor increased from 0.07 to 0.94 when the pressure was increased from 1.0 to 4.0 Pa, as shown in Figure 3f.^[^ ^121^ ^]^ With the transformation from (015) to (00*l*), the ratio of Bi:Te in the as‐deposited thin film was close to 2:3, and the grain size in the thin film was affected by sputtering pressure. Under 1.0 Pa or 2.0 Pa sputtering pressures, the average grain size was ≈400 nm, and the grains showed loose and rough features. With increasing sputtering pressure, the grain sizes were gradually reduced to ≈350 and ≈300 nm, and the thickness of the thin film was reduced from ≈1100 to ≈450 nm.^[^ ^121^, ^122^ ^]^ Bi_2_Te_3_ thin films fabricated under a 3.0 Pa sputtering pressure exhibited the highest *S* ^2^ *σ* of 21 μW cm^−1^ K^−2^ at room temperature. However, although the thin film fabricated under 4.0 Pa was optimized in the preferred orientation, the thickness and *σ* of this film was suppressed during sputtering, which seriously reduced the overall performance.^[^ ^121^ ^]^

The sputtering pressure affects the growth orientation and grain size of other thermoelectric thin films such as Sb_2_Te_3_. Sb_2_Te_3_ has been sputtered on polyimide (PI) films under sputtering pressures of 1.0, 2.0, 3.0, and 4.0 Pa with a chamber temperature of 473 K.^[^ ^123^ ^]^ When the sputtering pressure was 1.0 Pa, the preferred orientation for Sb_2_Te_3_ thin film growth was (104). With increasing sputtering pressure, the (104) peak gradually weakened and disappeared at a sputtering pressure of 3.0 Pa. When the sputtering pressure was 4.0 Pa, the preferred orientation was (015).^[^ ^123^ ^]^ In addition, higher sputtering pressure induced smaller grain sizes. The porosity of the as‐fabricated thin film fabricated at 1.0 Pa was very high (13.0%), while that of the film fabricated at 4.0 Pa was only 2.0%. Such a reduction in porosity was favorable for carrier transport.^[^ ^123^ ^]^ The film produced at 4.0 Pa exhibited the highest *S* ^2^ *σ* of >6 μW cm^−1^ K^−2^ at room temperature.^[^ ^123^ ^]^

### 3.3 Substrates

Rational selection of substrates for deposition is important for high‐quality magnetron sputtering, which directly impacts the compositions and properties of the as‐deposited thin films. To evaluate the influences of different substrates on the as‐sputtered thermoelectric thin films, amorphous glass, polycrystalline alumina, single‐crystalline sapphire, and organic PI were employed as substrates for depositing Bi_2_Te_3_ thin films.^[^ ^61^ ^]^ Figures 3g–j shows Bi_2_Te_3_ thin films deposited on glass, alumina, sapphire, and PI under different annealing conditions, from which the growths of Bi_2_Te_3_ on inorganic substrates were similar, that is, Bi_2_Te_3_ grains were gradually grown and merged together to form a solid phase.^[^ ^61^ ^]^ In contrast, the microstructure of the Bi_2_Te_3_ thin film deposited on the PI substrate exhibited a nanoparticle‐like morphology, which may be ascribed to the shrinkage of the PI substrate during the fabrication process. Figure 3k plots the Te ratio of Bi_2_Te_3_ deposited on the four substrates under three different conditions, namely, nonsubstrate heating (Condition 1), heating (Condition 2), and annealing (Condition 3). Under the conditions of heating and annealing (Conditions 2&3), the substrate had a large influence on the sputtering ratio of Bi and Te.^[^ ^61^ ^]^ An improved *σ* could be found in the films deposited on glass, alumina, and sapphire substrates, while the PI substrate induced a small average grain size, which resulted in degradation of *σ*. Figure 3l plots the *S* ^2^ *σ* of Bi_2_Te_3_ thin films deposited on the four substrates under three conditions, from which the thin films without heating or annealing treatments exhibited relatively low *S* ^2^ *σ*. A high *S* ^2^ *σ* was 27.3 μW cm^−1^ K^−2^ at room temperature by depositing on Bi_2_Te_3_ glass substrates, which was much higher than that of the other three substrates.^[^ ^61^ ^]^ In addition to the aforementioned substrates, cellulose fibers (CFs) can be employed as substrates.^[^ ^58^ ^]^ The specific features of CFs including high porosity and irregular pore orientation allowed the deposition of thinner thin films and prohibited the cracking of the TEDs.^[^ ^58^ ^]^ In addition, CFs possess the features of low *κ* and insulation, which inhibited thermal short circuits and current short circuits and were important for fabricating in‐plane configuration thin‐film TEDs.^[^ ^58^ ^]^ However, the performance of TEDs fabricated based on CFs as substrates was relatively low as reported, which needs to be further improved.

Because substrates can influence grain growth, a rationally selected substrate can realize the epitaxial growth of some thermoelectric thin films. For example, ScN is a semiconducting transition metal nitride, which can be synthesized by DC reactive magnetron sputtering as a high‐temperature thermoelectric material.^[^ ^80^ ^]^ It was reported that when applying MgO as a substrate, at a sputtering pressure of 0.27 Pa and a sputtering temperature of 1103 ± 20 K, the ScN can directly form nucleation in the upper layer of MgO, and form a mound‐like morphology, as shown in the SEM image in Figure 3m.^[^ ^80^ ^]^ As for the thermoelectric performance, ScN thin film exhibited the highest *S* ^2^ *σ* of 35 μW cm^−1^ K^−2^ at 600 K. Figure 3n exhibits the practical and theoretical *S* ^2^ *σ* of the ScN thin film as a function of temperature, which illustrated that the *S* ^2^ *σ* of the thin film did not deviate from the theoretical value. Compared with the magnetron sputtering method, plasma‐assisted molecular beam epitaxy can be used to deposit epitaxially grown ScN films, but *S* ^2^ *σ* was 23 μW cm^−1^ K^−2^ and the overall performance was worse than that deposited by magnetron sputtering since the presence of impurities such as oxygen in the Sc source of the process induced an excessive carrier concentration.^[^ ^124^ ^]^

### 3.4 Optimizing Sputtering Temperature

**Figure 4:**
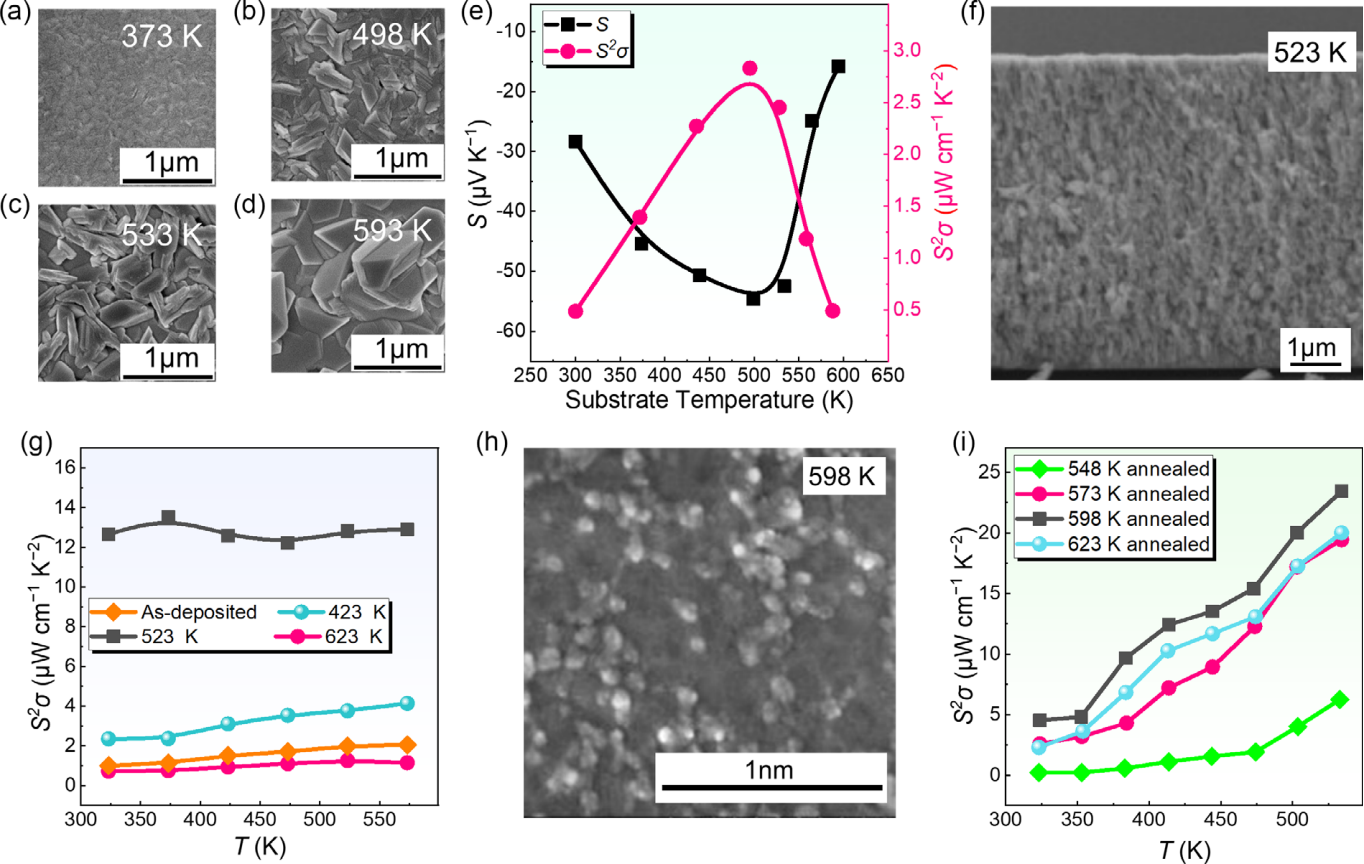
Effect of sputtering temperature and annealing temperature on magnetron‐sputtered films. a–d) SEM images of Bi_2_Te_3_ films deposited at different substrate temperatures namely 373, 498, 533, and 593 K, respectively. e) *S* and *S* ^2^ *σ* of Bi_2_Te_3_ films as functions of substrate temperatures. a–e) Reproduced with permission.^[^ ^125^ ^]^ Copyright 2006, Elsevier. f) Cross‐sectional SEM image of Bi_2_Te_3_ film annealed at 523 K. g) Temperature‐dependent *S* ^2^ *σ* of Bi_2_Te_3_ films annealed at different temperatures. f,g) Reproduced with permission.^[^ ^68^ ^]^ Copyright 2020, Elsevier. h) SEM image of ZnSb film annealed at 598 K. i) Temperature‐dependent *S* ^2^ *σ* of ZnSb films annealed at different temperatures. h,i) Reproduced with permission.^[^ ^70^ ^]^ Copyright 2014, Springer Nature.

In addition to the selection of substrates, the sputtering temperature can directly affect the structures and thermoelectric properties of the as‐deposited thin films, which is mainly derived from the fact that temperature can significantly influence the bonding as well as the crystalline process since the energy for bonding or crystalline can be directly provided by external thermal energy. **Figure** **4**a–d shows SEM images of Bi_2_Te_3_ films deposited at different substrate temperatures, namely, 373, 498, 533, and 593 K. When using high‐temperature‐resistant SiO_2_/Si as substrates, a low sputtering temperature could result in smooth and glossy surfaces of Bi_2_Te_3_ thin films without crystalline phases. On the contrary, with increasing sputtering temperature, the thin film surface gradually exhibited polycrystal features. In Figure 4a, the polycrystals did not exhibit an epitaxial growth behavior since SiO_2_/Si substrate was an amorphous oxide.^[^ ^125^ ^]^ With increasing sputtering temperature, the grain gradually grew with larger sizes, as shown in Figure 4b,c. When the sputtering temperature was achieved at 563 K, different types of grain structures appeared in the films, as shown in Figure 4d.^[^ ^125^, ^126^ ^]^ In terms of the performance, as shown in Figure 4e, when the sputtering temperature was 498 K, the maximum *S* and *S* ^2^ *σ* can be achieved with the values of ≈55 μV K^−1^ and 3 μW cm^−1^ K^−2^, respectively. In addition, the sputtering temperature may influence the transparency of some transparent materials such as CuI.^[^ ^127^ ^]^

### 3.5 Annealing

In addition to optimizing the pressure, type of substrate, and temperature of sputtering, other factors can significantly affect the final properties of thermoelectric thin films, such as annealing, hybridization, and doping effects. For example, the annealing process was reported to significantly influence the structure and performance of Bi_2_Te_3_ thin film deposited on Pi thin films.^[^ ^68^ ^]^ With increasing annealing temperature to 623 K, the Te ratio of Bi_2_Te_3_ was decreased from 59.02% to 57.38%, which could induce Te volatilization during the high temperature. The annealing process could break some chemical bonding of Bi_2_Te_3_, leading to the formation of Bi^3+^ and Te^2−^ along with Bi and Te single elements existing on the surface of the thin film.^[^ ^68^ ^]^ Figure 4f shows a cross‐sectional SEM image of magnetron‐sputtered Bi_2_Te_3_ thin film annealed under 523 K, which exhibited relatively large and uniform grain sizes. When the annealing temperature was increased, the films started to grow inward from the columnar structure, the crystallization was optimized, and the agglomeration phenomenon became less significant. However, high‐temperature annealing could induce cracks in the as‐fabricated thin films, attributed to the different expansion coefficients of the substrate and Bi_2_Te_3_.^[^ ^68^, ^128^, ^129^ ^]^ In terms of the thermoelectric performance, Figure 4g compares *S* ^2^ *σ* of Bi_2_Te_3_ films at different annealing temperatures, from which a maximum *S* ^2^ *σ* of 13.5 μW cm^−1^ K^−2^ could be achieved at an annealing temperature of 523 K.^[^ ^68^ ^]^ In addition to the annealing temperature, the annealing time can affect the stability and the thermoelectric performance of thin films. It was reported that by increasing the annealing time up to 5 h, magnetron‐sputtered Bi_2_Te_3_ thin film could exhibit better and more stable thermoelectric performance.^[^ ^130^ ^]^ However, the annealing time should be moderately reduced when the thin films were deposited on organic substrates since the organic substrates cannot withstand high temperatures for a long time.^[^ ^131^ ^]^

Sometimes, the annealing parameters may vary according to the types of thermoelectric materials. For example, ZnSb is a relatively cheap and nontoxic material that exhibits good thermoelectric performance at medium temperatures.^[^ ^70^ ^]^ It was reported that when the annealing time was 1 h and using different annealing temperatures at 473, 498, 523, 548, 573, 598, and 623 K, the as‐deposited films were insulated annealed at 473 and 498 K, which were not suitable for thermoelectric applications.^[^ ^70^ ^]^ With increasing the annealing temperature, the thin film exhibited an amorphous feature for the annealed at 523 K and blurred, ununiform, and irregular grains for the annealed at 548 K. This phenomenon indicated that the film crystallization became better, and the surface became smoother with increasing annealing temperature, as shown in Figure 4h. Figure 4i compares the *S* ^2^ *σ* of ZnSb films annealed at different temperatures. ZnSb thin film for the annealed at 598 K showed a maximum *S* ^2^ *σ* of 23.5 μW cm^−1^ K^−2^ at 533 K.^[^ ^70^ ^]^ Apart from magnetron sputtering, ZnSb can be fabricated by the screen‐printing method. The as‐fabricated ZnSb annealed at 853 K had a maximum *S* ^2^ *σ* of 10.6 μW cm^−1^ K^−2^, which was lower than magnetron sputtering‐made thin films.^[^ ^70^, ^132^ ^]^ This should be derived from the difference in practical compositions and structures developed by different fabrication processes.

### 3.6 Hybridization

Apart from the annealing treatment, hybridization is a good strategy for optimizing the thermoelectric performance of magnetron‐sputtered thin films. Hybridization can be described as introducing secondary materials (phases) with various dimensions into the as‐sputtered thin films, and these secondary materials usually have outstanding *S* or *σ* to optimize the thermoelectric properties of the overall hybrid thin film. The triggered energy filtering effect at the interfaces between different phases with different band structures can effectively filter the low‐energy carriers and in turn, improve the overall *S* but keep a high *σ*.^[^ ^2^, ^24^, ^29^, ^38^, ^57^, ^133^, ^134^, ^135^, ^136^, ^137^, ^138^ ^]^ Magnetron sputtering can fabricate such hybrid thin films either by cosputtering from different targets to form hybrid thin films with relatively uniformly distributed secondary phase, or directly sputtering the secondary layers on “substrate” thin films to form multilayered structures.

**Figure 5:**
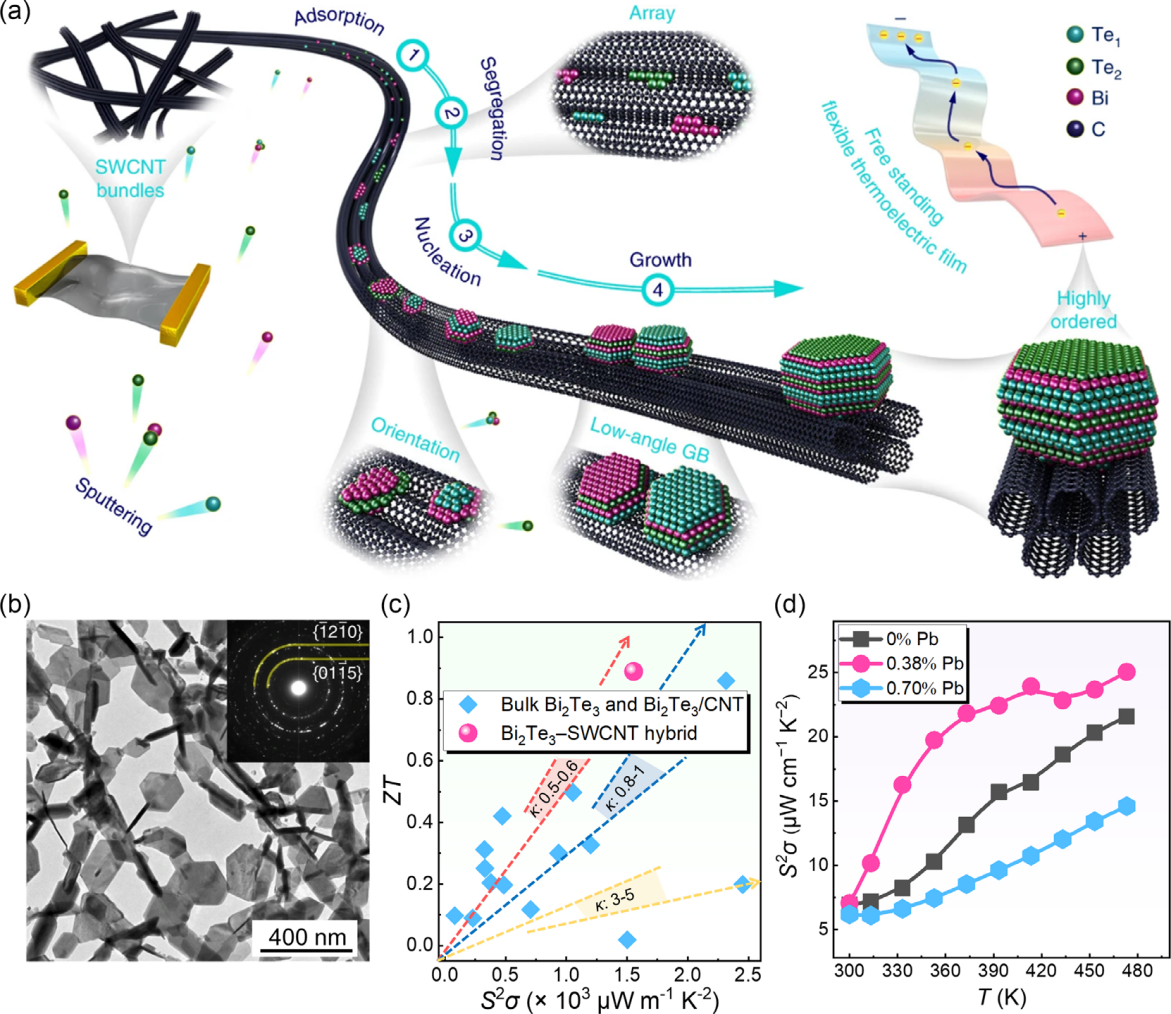
Effect of hybridization and doping on magnetron‐sputtered films. a) Schematic image of the highly (000*l*)‐textured Bi_2_Te_3_ nanocrystals grown on SWCNT bundles. b) TEM image of hybridized film after 120 s of deposition. The inset image shows the corresponding selected‐area diffraction pattern. c) The *S* ^2^ *σ* and *ZT* of reported conventional bulk Bi_2_Te_3_, Bi_2_Te_3_/CNT hybrid material, and Bi_2_Te_3_‐SWCNT hybrid material at room temperature. a–c) Reproduced with permission.^[^ ^64^ ^]^ Copyright 2018, Springer Nature. d) Temperature‐dependent *S* ^2^ *σ* of Bi_2_Te_3_ films doping with different Pb concentrations. Reproduced with permission.^[^ ^51^ ^]^ Copyright 2014, Elsevier. Here TEM refers to transmission electron microscopy, CNT refers to carbon nanotube, and SWCNT refers to single‐wall carbon nanotube.

Historically, carbon nanotubes (CNTs)/Bi_2_Te_3_ hybrid thin films were designed for the applications of hybrid thin films developed by magnetron sputtering. CNTs are promising flexible materials with high *σ* and flexibility.^[^ ^64^, ^139^, ^140^ ^]^ **Figure** **5**a shows a schematic image of highly (000*l*)‐textured Bi_2_Te_3_ nanocrystals grown on single‐wall carbon nanotube (SWCNT) bundles; here the alignment between Bi_2_Te_3_ *<* $\overset{¯}{1}2\overset{¯}{1}$0*>* and the SWCNT bundle groove/axis is good.^[^ ^64^ ^]^ During the sputtering process, Bi_2_Te_3_ was preferentially deposited in the grooves between SWCNTs, which provided an inducement for crystal orientations. Figure 5b illustrates a corresponding bright‐field transmission electron microscopy (TEM) image, which evidenced that Bi_2_Te_3_ prefers anisotropic growth parallel and perpendicular to the (000*l*) atomic plane.^[^ ^64^ ^]^ To obviously illustrate the high performance of the Bi_2_Te_3_‐SWCNT hybrid thin film, Figure 5c compares the room‐temperature *ZT* between conventional Bi_2_Te_3_, flexible Bi_2_Te_3_/CNT hybrid, and Bi_2_Te_3_‐SWCNT hybrid. The as‐fabricated Bi_2_Te_3_‐SWCNT hybrid material exhibit a lower *κ* and a higher *S* ^2^ *σ*, leading to better thermoelectric performance.^[^ ^64^ ^]^ The relatively low *κ* may be derived from its lower *κ* (mainly *κ*_l_) since this thin film possesses a variety of lattice distortions such as dislocations and interfaces between SWCNTs and Bi_2_Te_3_, which effectively scatter the phonons with different wavelengths.^[^ ^64^, ^141^, ^142^, ^143^ ^]^

### 3.7 Doping

Doping is an effective strategy for improving the overall thermoelectric performance of magnetron‐sputtered thin films. Generally, rational doping can further tune the electron carriers for n‐type thin films or hole carriers for p‐type thin films, breaking the trade‐off between *S* and *σ* and in turn optimizing *S* ^*2*^ *σ*.^[^ ^8^, ^144^, ^145^, ^146^, ^147^ ^]^ Sometimes doping has been used to slightly reduce the *n* and in turn improve the *S* to optimize *S* ^2^ *σ*. Besides, doping is beneficial for introducing point defects in the as‐fabricated thin films by introducing doping atoms with different radii, which can scatter short‐wavelength phonons and help suppress *κ*_l_ and *κ*. Therefore, doping has been widely applied during the magnetron sputtering of thermoelectric thin films.^[^ ^2^, ^8^, ^144^, ^145^ ^]^

Pb was commonly used as a dopant to regulate the hole carrier concentration in Bi_2_Te_3_‐based thin films.^[^ ^51^, ^148^, ^149^ ^]^ Pb‐doped Bi_2_Te_3_ thin films were prepared using a radio frequency (RF) magnetron sputtering technique,^[^ ^51^ ^]^ from which three different Pb doping concentrations were designed as 0%, 0.38%, and 0.7%. Figure 5d compares temperature‐dependent *S* ^2^ *σ* of Bi_2_Te_3_ films doped with different Pb concentrations, from which 0.38% Pb‐doped Bi_2_Te_3_ thin film exhibited the highest *S* ^2^ *σ* of 25 μW cm^−1^ K^−2^ at 473 K.^[^ ^51^ ^]^ It should be noted that a higher doping concentration (0.7%) resulted in the lowest performance. This should be attributed to the excess Pb, which resulted in a decreased *n* and suppressed carrier–carrier scattering.

**Figure 6:**
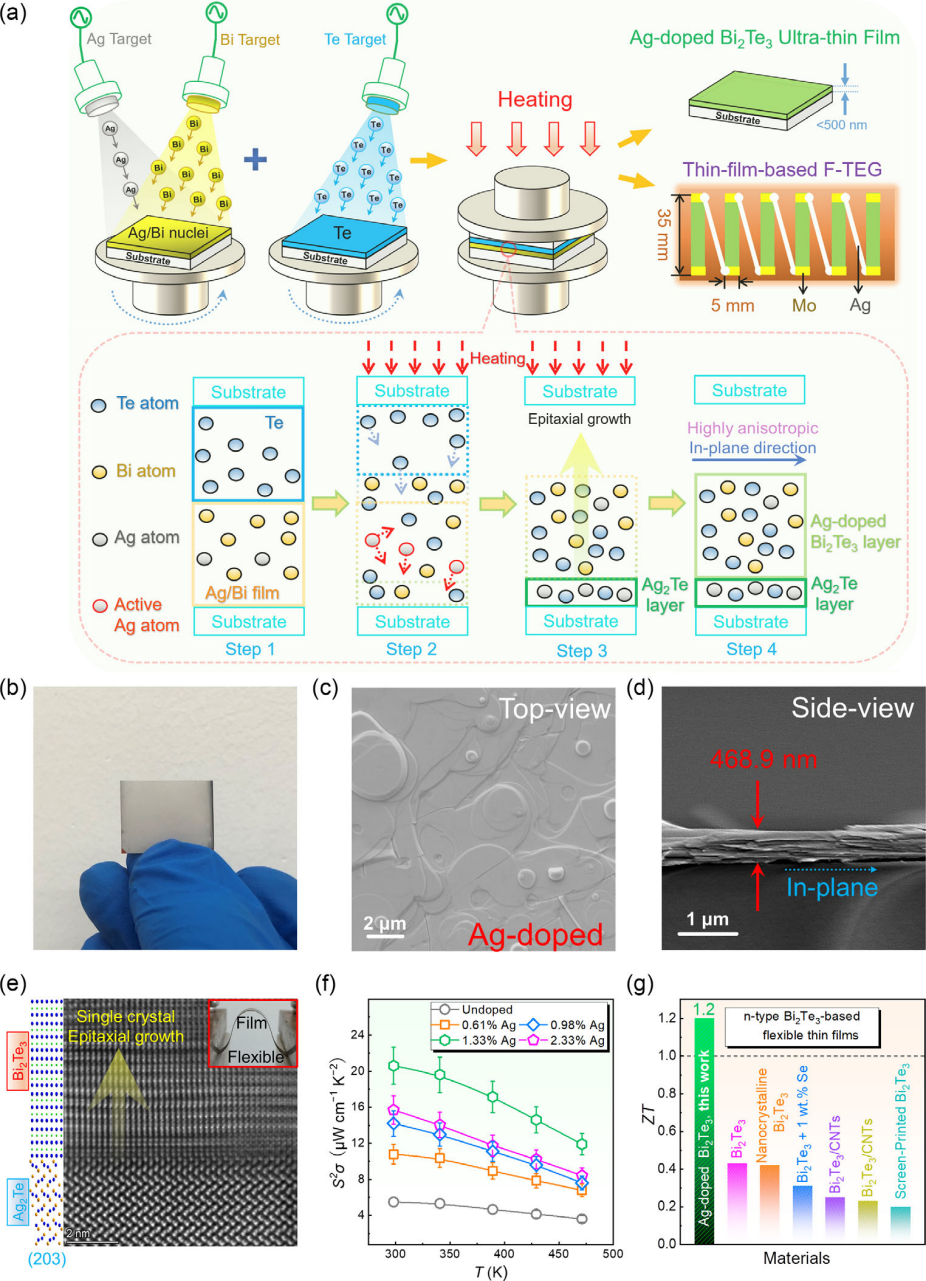
Ag‐doped Bi_2_Te_3_ films prepared by a combination of magneton sputtering and thermal diffusion reaction method. a) Schematic of fabricating Ag‐doped Bi_2_Te_3_ thin film prepared by magnetron sputtering and followed thermally induced close diffusion reaction. b) Photo of as‐achieved Ag‐doped Bi_2_Te_3_ thin film. c,d) Top‐ and side‐view SEM images of 1.33% Ag‐doped Bi_2_Te_3_ thin film. e) HAADF image of Ag‐doped Bi_2_Te_3_ thin film by Cs‐STEM along the [210] orientation. f) Temperature‐dependent *S* ^2^ *σ* of Ag‐doped Bi_2_Te_3_ films. g) Comparison of room‐temperature *ZT* between Ag‐doped Bi_2_Te_3_ thin film and other reported n‐type Bi_2_Te_3_‐based thin films. a–g) Reproduced with permission.^[^ ^8^ ^]^ Copyright 2022, Springer Nature.

In addition to cosputtering, doping can be performed by the thermal diffusion reaction method.^[^ ^8^, ^150^, ^151^ ^]^ For highly flexible Ag‐doped Bi_2_Te_3_ thin films, Bi was first sputtered on the PI substrate, during which Ag was cosputtered onto the PI substrate to prepare Ag/Bi cores.^[^ ^8^ ^]^ Te was simultaneously deposited on another PI substrate. The two films were then brought into close contact under a certain external pressure and heat treated at 623 K for 30 min in a vacuum stove, as shown in **Figure** **6**a. After heat treatment, the Ag‐doped Bi_2_Te_3_ films could be achieved as shown by the photo in Figure 6b.^[^ ^8^ ^]^ The microstructures and phase information of the 1.33% Ag‐doped‐Bi_2_Te_3_ thin films were characterized by SEM including the top view and side view, as shown in Figure 6c,d.^[^ ^8^ ^]^ The SEM results exhibited a layer‐by‐layer feature with ultrahigh anisotropy properties and a thickness of 468.9 nm. Apart from SEM images showing unique material structures, the high‐angle‐annular dark‐field (HAADF) image based on scanning transmission electron microscopy (STEM) along the [210] orientation also reveals the reasons for the as‐achieved high thermoelectric performance and flexibility.^[^ ^8^ ^]^ The spherical aberration‐corrected STEM (Cs‐STEM) image (Figure 6e) confirmed the nanostructure characteristics of the as‐prepared films, from which significant lattice distortions were found to be homogeneously distributed within the film, resulting in significant lattice strains with strong anisotropy.^[^ ^8^ ^]^ Such significant lattice distortions were caused by both interstitially doped and substitute‐doped Ag, which could effectively scatter phonons and suppress the *κ* of the film to 0.47 W m^−1^ K^−1^. Besides, as the Ag content was increased from 0% to 1.33%, the *σ* of the thin film can be significantly enhanced,^[^ ^8^ ^]^ leading to the highest *S* ^2^ *σ* of 20.59 μW cm^−1^ K^−2^ at room temperature, as shown in Figure 6f.^[^ ^8^ ^]^ Therefore, when the Ag content was 1.33%, the largest *ZT* of ≈1.2 could be achieved at room temperature, as shown in Figure 6g.^[^ ^8^ ^]^ Compared with other reported Bi_2_Te_3_‐based thermoelectric thin films (*ZT* < 0.5), such good performance enables Ag‐doped Bi_2_Te_3_ thin films to fabricate high‐performance flexible/wearable devices.^[^ ^8^ ^]^

Apart from Bi_2_Te_3_, doping can be achieved in selenides by magnetron sputtering. For example, Ag_2_Se is one of the good candidates among thermoelectric selenides due to its unique features of low‐cost, high performance, and ductility,^[^ ^152^ ^]^ which exhibit good prospects in near‐room‐temperature applications. To further improve the thermoelectric performance of Ag_2_Se‐based thin films, Cu can be employed as a dopant during the sputtering process. Generally, Cu was equivalently doped on Ag sites, which can form an isoelectronic hole trap since Cu had lower electronegativity than that of Ag.^[^ ^145^ ^]^ Meanwhile, with increasing the Cu‐doped concentration, part of Cu occupied Ag vacancies or gap positions with positive monovalent as donors to provide more electrons.^[^ ^145^, ^153^ ^]^ In terms of the sputtering process, the ratio of Cu during the sputtering process could be controlled by RF sputtering at different power levels. At a low Cu‐doped concentration, doped Ag_2_Se exhibited an obvious *c*‐axis orientation.^[^ ^145^ ^]^ With increasing doping level, (013) and (014) orientations became obvious. As for the thermoelectric performance, with increasing Cu content, *n* increased from 3.8 × 10^18^ to 9.9 × 10^18^ cm^−3^, but carrier mobility *μ* reduced from 1100 to 650 cm^2^ V^−1^ s^−1^. When employing 12 W sputtering power to dope Cu, the best *S* ^2^ *σ* of 20.8 μW cm^−1^ K^−2^ can be achieved at room temperature.^[^ ^145^ ^]^ Apart from the magnetron sputtering method, Ag_2_Se‐based thermoelectric thin films can be fabricated by other methods such as vacuum‐assisted filtration^[^ ^48^ ^]^ and pulsed laser deposition.^[^ ^145^, ^154^ ^]^ However, these thin films exhibited lower *μ* than those made of magnetron sputtering. This should be because magnetron sputtering often leads to better crystallization for inorganic thin films.^[^ ^145^ ^]^

### 3.8 Pulsed Magnetron Sputtering

For thermoelectric selenide‐based thin films, the Se element often exhibited difficulties in sputtering since Se is easy to be volatile at high temperatures. Therefore, excessive Se may be employed during the sputtering process. However, these Se may directly grow on other target and chamber walls, which may induce target and chamber contamination.^[^ ^85^ ^]^ A splitter valve can be employed to address the problem, which is called pulsed magnetron sputtering. Compared with conventional magnetron sputtering, pulsed magnetron sputtering can achieve alternate deposition of materials, which allows fine control of the film chemistry and fast growth rates. Pulsed magnetron sputtering is commonly used to produce oxide coatings by reacting metal targets with oxygen atmosphere. However, it can also be used to prepare selenide coatings using selenium vapor instead of oxygen.^[^ ^60^ ^]^ It was reported that pulsed magnetron sputtering was employed to fabricate Cu_2_Se thin films.^[^ ^85^ ^]^ These Cu_2_Se thin films exhibit good and stable performance without any annealing process, which means the entire sputtering process can be realized at room temperature.^[^ ^85^ ^]^ Cu_2_Se thin films made by pulsed magnetron sputtering prefer to grow into columnar crystals with a thickness of 733 nm, and there were only a few hexagonal nanoplates presented on the surface. The best *S* ^2^ *σ* and *ZT* were 11 μW cm^−1^ K^−2^ and 0.4 at room temperature when the Cu/Se ratio was 2.^[^ ^85^ ^]^ Apart from magnetron sputtering, Cu_2_Se can be fabricated by vacuum filtration and cold pressing.^[^ ^155^ ^]^ A Cu_2_Se/poly(3,4‐ethylenedioxythiophene) polystyrene sulfonate (PEDOT: PSS) hybrid material was fabricated by this method, which allowed the as‐designed thin film good flexibility. However, the *S* ^2^ *σ* of this thin film was 8.2 μW cm^−1^ K^−2^ which was lower than that made by magnetron sputtering.^[^ ^85^, ^155^ ^]^ Such *S* ^2^ *σ* reduction could be derived from the incorporation of organic components.

Ag_2_Se thin film was reported to be fabricated by pulsed magnetron sputtering. According to the splitter valve, the ratio of Ag/Se can be controlled to ≈2, which contributed to *S* ^2^ *σ* of 24.4 μW cm^−1^ K^−2^ in the as‐fabricated thin film at room temperature.^[^ ^96^ ^]^ When the test temperature was increased to ≈376 K, *S* ^2^ *σ* could be further increased to 46.5 μW cm^−1^ K^−2^, which resulted from a relatively high *S*.^[^ ^96^ ^]^ However, when the temperature exceeded ≈407 K, Ag_2_Se would convert from the rhomboidal β phase to the superionic cubic α phase. During the phase transition, the *S* sharply decreased, which seriously suppressed the *S* ^2^ *σ* to 5 μW cm^−1^ K^−2^.^[^ ^96^ ^]^ Apart from pulsed magnetron sputtering, Ag_2_Se was reported to be made by the coevaporation method,^[^ ^55^ ^]^ which showed *S* ^2^ *σ* of 20.51 μW cm^−1^ K^−2^ at room temperature. Such a value was lower than that made of the pulsed magnetron sputtering method since the latter one fabricated the thin film with a denser structure.^[^ ^55^ ^]^

## 4 Mechanical Performance

### 4.1 Flexibility and Normalized Resistance

**Figure 7:**
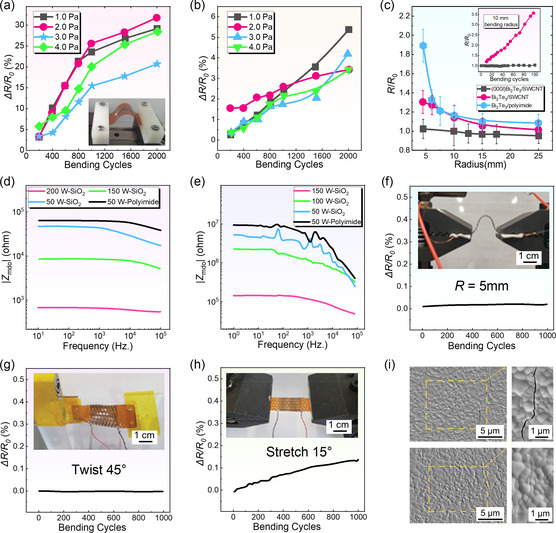
The degree of change in electrical resistivity after bending the films under different preparation conditions. a,b) Normalized resistance Δ*R*/*R*_0_ of magnetron‐sputtered Bi_2_Te_3_ thin films prepared under different sputtering pressures without (a) and with (b) a PDMS substrate at a bending radius of 7 mm for 400, 800, 1200, 1600, and 2000 cycles. Here *R*_0_ is the initial electrical resistance and Δ*R* is the difference between the electrical resistance after bending and *R*_0_. A photograph of Bi_2_Te_3_ film during the bending test is shown as inset in (a). a,b) Reproduced with permission.^[^ ^121^ ^]^ Copyright 2019, Elsevier. c) *R/R*_*0*_ of different types of Bi_2_Te_3_ thin films bent at different bending radius. Inset: Bending results of the (000*l*)‐textured hybrid and Bi_2_Te_3_/polyimide sample. Here *R* is the postbending electrical resistance. Reproduced with permission.^[^ ^64^ ^]^ Copyright 2018, Springer Nature. d,e) The electrical resistance of thin films as a function of frequency for Bi_2_Te_3_ (d) and Sb_2_Te_3_ (e). d,e) Reproduced with permission.^[^ ^156^ ^]^ Copyright 2021, Springer Nature. f–h) Δ*R*/*R*_0_ of TEDs integrated by a heuristic design in bending, twisting, and stretching conditions. Corresponding photos for Δ*R*/*R*_0_ testing are provided as insets. f–h) Reproduced with permission.^[^ ^157^ ^]^ Copyright 2021, Wiley‐VCH. i) SEM images of Ag_2_Se thin film in bending state (top) and self‐healing state (bottom). Reproduced with permission.^[^ ^145^ ^]^ Copyright 2022, Elsevier.

Compared with conventional bulk thermoelectric materials, ductility and flexibility are two advantages of thin‐film‐based thermoelectric materials. Generally, the flexibility of the thin film can be evaluated by performance stability during bending by different bending radii and bending cycles, which can be expressed by normalized resistance Δ*R*/*R*_0_, where Δ*R* is the difference in electrical resistance before and after bending, and *R*_0_ is the initial electrical resistance. To suppress the Δ*R*/*R*_0_ of thin‐film‐based thermoelectric materials, one strategy is employing flexible encapsulation or substrate to cover the surface or support the thermoelectric thin films to realize certain flexibility. Polydimethylsiloxane (PDMS) is a good candidate for this application, which has the features of good biocompatibility, chemical, and mechanical stability and is expected for encapsulation. It was reported that PDMS can optimize the mechanical properties of the thermoelectric thin film.^[^ ^121^ ^]^ To be specific, the Bi_2_Te_3_ thin films were sputtered on PI substrates with different sputtering pressures to evaluate the Δ*R*/*R*_0_ of the as‐fabricated thin film.^[^ ^121^ ^]^ **Figure** **7**a exhibits the Δ*R*/*R*_0_ of these thermoelectric thin films under different bending cycles with a bending radius of 7 mm. When the bending cycle was increased to 2000 times, the Δ*R*/*R*_0_ was increased to ≈30%, which seriously reduced the *σ.* ^[^ ^121^ ^]^ However, after employing PDMS as the encapsulation, Δ*R*/*R*_0_ increased by only <5% after 2000 times bending a bending radius of 7 mm, as shown in Figure 7b. When the bending ratio was decreased to 5 mm, the Δ*R*/*R*_0_ still exhibited no obvious change. Considering that a 5 mm bending radius was equivalent to the finger radius, using PDMS to cover thermoelectric thin film should satisfy broad practical applications.^[^ ^121^ ^]^

Rational selection of different types of flexible substrates and thin‐film orientations can further influence the flexibility of the films.^[^ ^64^ ^]^ To compare the differences in the flexibility, dense Bi_2_Te_3_/PI films, as well as (000*l*)‐oriented and non‐(000*l*)‐oriented Bi_2_Te_3_‐SWCNT hybrid films, were prepared using the same sputtering conditions.^[^ ^64^ ^]^ The change in electrical resistance is expressed as *R*/*R*_0_, where *R* is the post‐bending electrical resistance, as shown in Figure 7c,^[^ ^64^ ^]^ and the inset exhibits bending results of the (000*l*)‐textured hybrid and Bi_2_Te_3_/polyimide sample. Compared with the single‐phase Bi_2_Te_3_ film on the PI substrate, the non‐(000*l*) oriented Bi_2_Te_3_/SWCNT hybrid film has better flexibility due to the flexible SWCNTs as supporters and the porous structure of the film, which plays significant roles in boosting the overall flexibility during bending. Among the three films, the hybrid film with (000*l*) orientation exhibited the best flexibility. After 100 bending cycles at a bending radius of 10 mm, the *R*/*R*_0_ was increased by 3%; thus, the hybrid film has excellent mechanical flexibility. Besides, the *S* and *κ* did not significantly change after bending, and no crack was observed on the film surface.^[^ ^64^ ^]^

Besides, tuning the sputtering power can influence flexibility and electrical resistance. Generally, Bi_2_Te_3_ and Sb_2_Te_3_ thin films possess different flexibilities. Different sputtering powers resulted in different thermal stress; therefore, the microcracks of the as‐fabricated thin films are different.^[^ ^156^ ^]^ Figure 7d,e shows the electrical resistance of thin films as a function of frequency for Bi_2_Te_3_ and Sb_2_Te_3_, respectively. With increasing sputtering power, two Bode magnitude plots exhibited that the electrical resistivity *ρ* of the prepared films decreased.^[^ ^156^ ^]^ However, the thin films fabricated under low sputtering power exhibited relatively low thermoelectric performance since polycrystals usually occurred at low sputtering power.^[^ ^156^ ^]^

Apart from the influence of magnetron sputtering, the practical environment may influence Δ*R*/*R*_0_. 54 pairs of Bi_2_Te_3_ and Sb_2_Te_3_ thermoelectric thin films deposited by magnetron sputtering are integrated together by a kirigami‐inspired design, which can easily transfer from 2D thin‐film type to 3D adjustable architecture and exhibit good stability.^[^ ^157^ ^]^ To be specific, Δ*R*/*R*_0_ may be influenced by practical environment, such as bending, twisting, and stretching. Figure 7f–h shows the Δ*R*/*R*_0_ of TEDs integrated by a heuristic design in bending, twisting, and stretching conditions, and corresponding photos for Δ*R*/*R*_0_ testing are provided as insets. The innovative structure design for the device exhibits good stability. After 1000 bending cycles with a bending radius of 5 mm (Figure 7f), Δ*R*/*R*_0_ was only increased by ≈2%. Under 45° twisting deformation with 1000 times repeating, the change of Δ*R*/*R*_0_ was of ≈1%.^[^ ^157^ ^]^ However, when such a design faced stretch, Δ*R*/*R*_0_ was increased by ≈13%, which may result from the microcracks under such stretching. Therefore, rational design is of significance for devices composed of sputtered thin films.^[^ ^157^ ^]^ More discussions about the device designs based on magnetron sputtering will be provided in Section 5.

When flexible substrates were used such as PI, the flexibility can be improved to a certain extent since the cracks between large grains can easily merge during bending.^[^ ^145^ ^]^ Therefore, fewer grain boundaries lead to higher flexibility when using flexible substrates. It was reported that after 1000 bending cycles, a 4% Δ*R*/*R*_0_ resulted in Ag_2_Se thin film. However, after placing the film by 1 h, the Δ*R*/*R*_0_ was reduced. To further investigate the mechanism of electrical resistance recovery, the microcracks generation and recovery were recorded by SEM, as shown in Figure 7i,^[^ ^145^ ^]^ from which SEM images of Ag_2_Se thin film in bending state (up) and self‐healing state (down) are both provided. By a bending radius of 5 mm, it was observed that the films produced microcracks due to tensile stress. After restoring the film to its normal state and leaving it for 10 h, it was found that the microcracks on the film tended to vanish.^[^ ^145^ ^]^ During this recovery process, the small cracks on the film disappeared, indicating that the flexibility of inorganic thermoelectric film should be improved. Such a unique phenomenon was simultaneously caused by the flexible substrate used and the large grain sizes of the Ag_2_Se thin film. However, in other thermoelectric thin films such as Bi_2_Te_3_, such a phenomenon is difficult to observe. Therefore, deeper understanding is required to explain the fundamentals for such an interesting phenomenon.

### 4.2 Other Mechanical Properties

**Figure 8:**
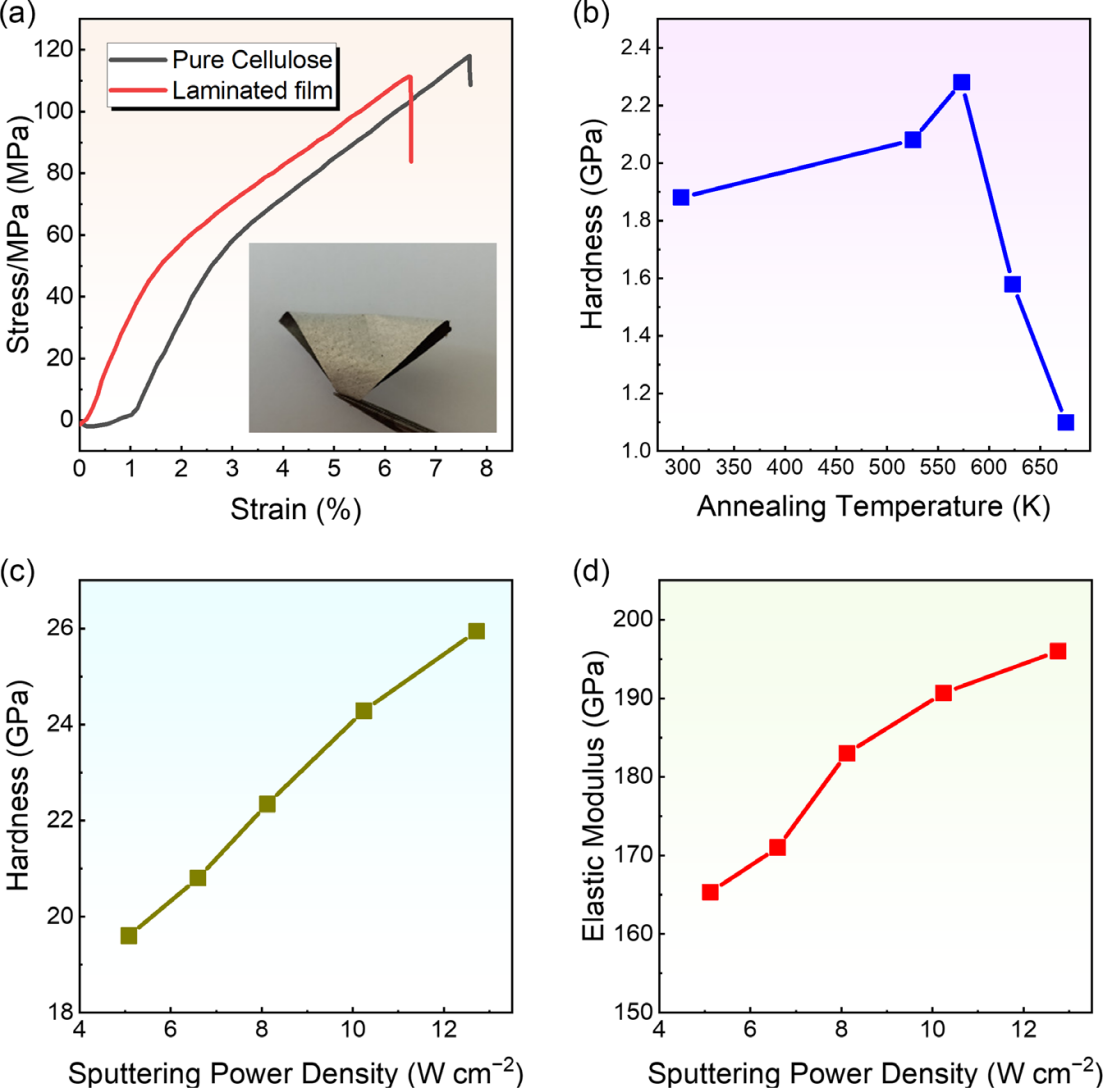
Mechanical properties of the films under different preparation conditions. a) Tensile stress–strain curves of laminated and pure cellulose films. The inset image shows the photo of the bent laminated film. Reproduced with permission.^[^ ^158^ ^]^ Copyright 2021, American Chemical Society. b) Hardness of Bi_2_Te_3_ films as a function of annealing temperature. Adapted under the terms of the CC‐BY Creative Commons Attribution 3.0 Unported license (https://creativecommons.org/licenses/by/3.0).^[^ ^159^ ^]^ Copyright 2017 Vietnam Academy of Science & Technology. c,d) Plots of hardness (c) and elastic modulus (d) as functions of sputtering power density. Reproduced with permission.^[^ ^160^ ^]^ Copyright 2018, Elsevier.

Apart from Δ*R*/*R*_0_, the flexibility of the thermoelectric thin film can be estimated by some other criteria, such as elongation at break, stress–strain, and elastic modulus. **Figure** **8**a compares tensile stress–strain curves of laminated and pure cellulose films. Compared with the pure cellulose with an elongation at a break of 6.47% and tensile stress of 104.7 MPa, the MnO_2_ nanowires@Ag/cellulose‐laminated membranes had a higher elongation at a break of 7.61% with a higher tensile stress of 110.5 MPa. Therefore, laminate technology can influence strain stress, and MnO_2_ nanowire@Ag/cellulose laminate films are expected to be used for thermal management applications, which require good mechanical properties.^[^ ^158^ ^]^ Such good mechanical properties can be attributed to the fiber structures of building blocks and the interface stability of laminated membranes.^[^ ^158^ ^]^

In addition to the influence of material compositions, the annealing temperature during the magnetron sputtering process can affect the mechanical properties of thermoelectric thin films. Figure 8b shows the measured hardness of Bi_2_Te_3_ films as a function of annealing temperature. The hardness of Bi_2_Te_3_ thin film was increased to 2.30 GPa at an annealing temperature of 573 K.^[^ ^159^ ^]^ Then the hardness sharply decreased during the annealing temperature of 573–673 K, which can be attributed to the escaped Te from Bi_2_Te_3_, and the density of the thin film was seriously affected.^[^ ^159^ ^]^ Sputtering power is an important factor to influence the mechanical properties of thermoelectric thin films.^[^ ^160^ ^]^ Figure 8c,d shows hardness and elastic modulus as functions of sputtering power density, respectively. According to Figure 8c,d, the hardness and elastic modulus of TiB_2_ thermoelectric thin film were influenced by sputtering power density.^[^ ^160^ ^]^ When the sputtering power density was increased from 4 to 13 W cm^−2^, the hardness and elastic modulus increased from 19.5 and 165.2 GPa to 26.6 and 196.8 GPa.^[^ ^160^ ^]^ The reason for this situation is that higher sputtering power density induced higher atom mobility and made the structure of the thin film denser, which increased the hardness and elastic modulus.^[^ ^160^ ^]^

## 5 Device Design

**Figure 9:**
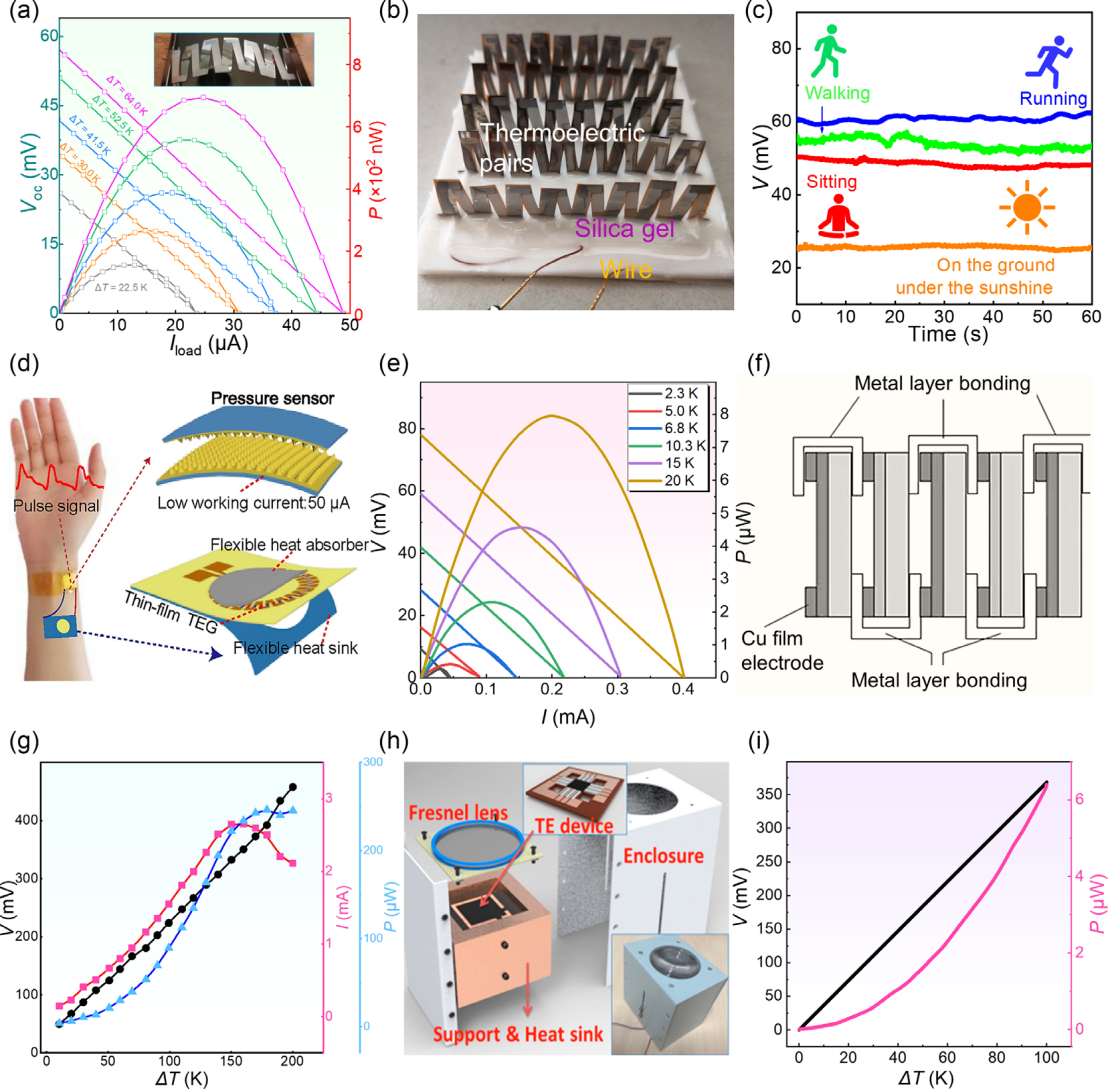
Device performance of magnetron‐sputtered thin films. a) *V*–*I* and *P*–*I* curves at different temperature differences of Ag‐doped Bi_2_Te_3_ thin‐film‐fabricated prototype TEG. Inset: A photo of the prototype. b) Photo of an optimized wearable TEG. c) Performance when the as‐fabricated wearable TEG is worn at different motion states. a–c) Reproduced with permission.^[^ ^8^ ^]^ Copyright 2022, Springer Nature. d) Schematic diagram of a flexible film‐based TED integrated with a pressure sensor. e) Voltage–current (*V*–*I*) and power–current (*P*–*I*) curves at different temperature differences (Δ*T*s). d,e) Reproduced with permission.^[^ ^161^ ^]^ Copyright 2020, Elsevier. f) Schematic diagram of a film‐based TED connected by a metal layer. g) Measured *V*, *I*, and *P* of the TED as a function of Δ*T* when the cold‐side temperature *T*_c_ was 300 K. f,g) Reproduced with permission.^[^ ^162^ ^]^ Copyright 2015, American Institute of Physics. h) Schematic diagram of a film‐based TED with an integrated concentrator. i) Corresponding measured *V* and *P* of the TED as a function of Δ*T*. h,i) Reproduced with permission.^[^ ^163^ ^]^ Copyright 2020, Elsevier.

One of the goals for thermoelectric material optimization is applying these materials to practical devices for power generation and refrigeration.^[^ ^8^ ^]^ Therefore, designs and fabrications of TEDs are important. Magnetron sputtering is one of useful techniques to fabricate TEDs. Especially, flexible inorganic thermoelectric films and their devices can be used in many different application environments.^[^ ^8^ ^]^ It was reported that when employing Ag‐doped Bi_2_Te_3_‐based thin film as n‐type and Sb_2_Te_3_ thin film as p‐type elements to fabricate horizontally structured prototype TEG, the maximum *P* can achieve 700 nW at the Δ*T* of 64 K (**Figure** **9**a), and the power density *ω* can achieve 2065.8 μW cm^−2^.^[^ ^8^ ^]^ According to such good performance, these materials were fabricated into an optimized wearable TEG (Figure 9b), which can establish of good and stable Δ*T* between hot and cold sides. To better demonstrate the performance and stability of the as‐fabricated wearable TEG, Figure 9c shows the working state of the TEG when that was worn on a human body with different motions. Apart from this, Bi_2_Te_3_ and Sb_2_Te_3_ were reported to be used as other flexible TEDs.^[^ ^8^ ^]^ It was reported that 25‐pair Bi_2_Te_3_ and Sb_2_Te_3_ thermoelectric legs were sputtered on a PI substrate to form a TED, which can be employed for powering medical monitors.^[^ ^161^ ^]^ Figure 9d shows the schematic diagram of a flexible film‐based TED integrated with a pressure sensor. This TED was composed of the Cu films as electrodes, the polydimethylsiloxane/boron nitride (PDMS/BN) composite films as encapsulation, and the hydrogel as a heat sink. Figure 9e shows the plotted open‐circuit voltage*–*current (*V–I*) and output power*–*current (*P–I*) curves.^[^ ^161^ ^]^ When the Δ*T* was 20 K, the maximum *P* was of 7.9 μW. As for the encapsulation, PDMS had the features of safety and flexibility, which guaranteed reliability when in contact with the human body for a long time. BN had the properties of high *σ* and low *κ*, which was imported into PDMS as the filler and helped to transfer the body heat to the hot side of TED.^[^ ^161^ ^]^ Considering the thin PDMS/BN encapsulation may result in a high temperature on the cold side at the same time, the heat sink is a compulsory for the as‐fabricated TED. Hydrogel heat sinks had a 3D network structure, which can make the contained H_2_O fast evaporate to reduce the temperature of the cold side.^[^ ^161^ ^]^ With the hydrogel as a heat sink, the TED can run continuously for 8 h and maintain a high voltage *V* of ≈15 mV. As for the flexibility of the as‐fabricated TED, PI substrate enabled the TED good flexibility with a <5% change of *ρ* and *V* after 1200 bending cycles. Owing to the good flexibility and the unique design, such a TED can be potentially employed on medical equipment.^[^ ^9^ ^]^

Compared with bulk‐based TEDs, thin‐film‐based TEDs have a faster response time,^[^ ^162^ ^]^ which can be employed in some heat recovery applications. For example, Zn–Sb thin films exhibited good thermoelectric performance at 433–473 K, which may be employed for collecting the waste heat of vehicle exhaust pipes.^[^ ^162^ ^]^ It was reported that Al‐doped ZnO (p‐type) and ZnSb (n‐type) were individually sputtered on Kapton substrate with 100 W DC sputtering powers. To estimate the performance of the ZnO/ZnSb p–n junction, the highest *V* of 46.64 mV can be achieved at a Δ*T* of 476 K.^[^ ^162^ ^]^ However, when the Δ*T* was continuously increased, the Kapton substrate may have serious deformation and result in electrode dislodgement and poor contact. When employing 10 p–n pairs to fabricate the thin‐film TED (Figure 9f), the maximum *P* was 246.3 μW at a Δ*T* of 180 K (Figure 9g), which was expected for recycling the waste heat of vehicles.^[^ ^162^ ^]^

In addition to waste heat recovery, thermoelectric thin films and their TEDs can be employed with other functional technologies, such as photovoltaic applications.^[^ ^163^ ^]^ When TEDs were combined with spectrally selective coatings, solar energy can be converted into electricity. According to magnetron sputtering, Bi_0.5_Sb_1.5_Te_3_ and Bi_0.5_Te_2.7_Se_0.3_ were sputtered on substrates under sputtering pressures of 0.5 Pa, sputtering powers of 30 W, and sputtering temperatures of 623 K. Cu/Ni materials were employed as electrodes for improving the contact.^[^ ^163^, ^164^ ^]^ As for the integrated devices, Figure 9h shows a schematic of the concentrator/TED, which contains a shell with a Fresnel lens and focal length mounted on top. The interior of the shell has a hollow copper heat sink, which can be adjusted in height in the shell. The cold side of TED made of 12 pairs of thermoelectric legs was placed on the heat sink with the outer edges tightly pressed by a square ring block and the rest of the TED suspended. In addition, a heat absorber with light absorbance of 0.96 and emissivity of 0.05 was set at the middle range of TED.^[^ ^163^ ^]^ In terms of the working principle, when the as‐integrated devices were exposed to the sunlight, the light is focused on the spectrally selective coating through the Fresnel lens, which leads to the temperature increase on the hot side. To illustrate the differences between integrated devices and pure TED, the illumination intensity was set as 30 mW cm^−2^ to compare the performance of these devices.^[^ ^163^ ^]^ Conventional TEDs exhibited a *V* of 16.5 mV, while the integrated device showed a *V* of 150 mV. The concentrator can accumulate heat at the core of the integrated device, which can produce a higher Δ*T.* ^[^ ^163^ ^]^ Figure 9i illustrates the relationship between the performance of devices and Δ*T.* At standard solar radiation, the Δ*T* in the as‐integrated device can reach 373 K, leading to a *V* of 375 mV and a *P* of 6.5 μW.^[^ ^163^ ^]^ The unbalanced cross‐sectional area may influence the performance of the as‐fabricated device, while increasing the thickness of the thermoelectric thin films can effectively optimize the device performance.^[^ ^163^ ^]^ As for the durability of the as‐fabricated device, the peak performance can be achieved after 10 min of operation of the device and can maintain the performance for 1 h. Therefore, the device meets the needs of small‐scale solar energy collection and has the potential for light‐sensing applications.^[^ ^163^ ^]^

## 6 Conclusions, Challenges, and Outlooks

**Figure 10:**
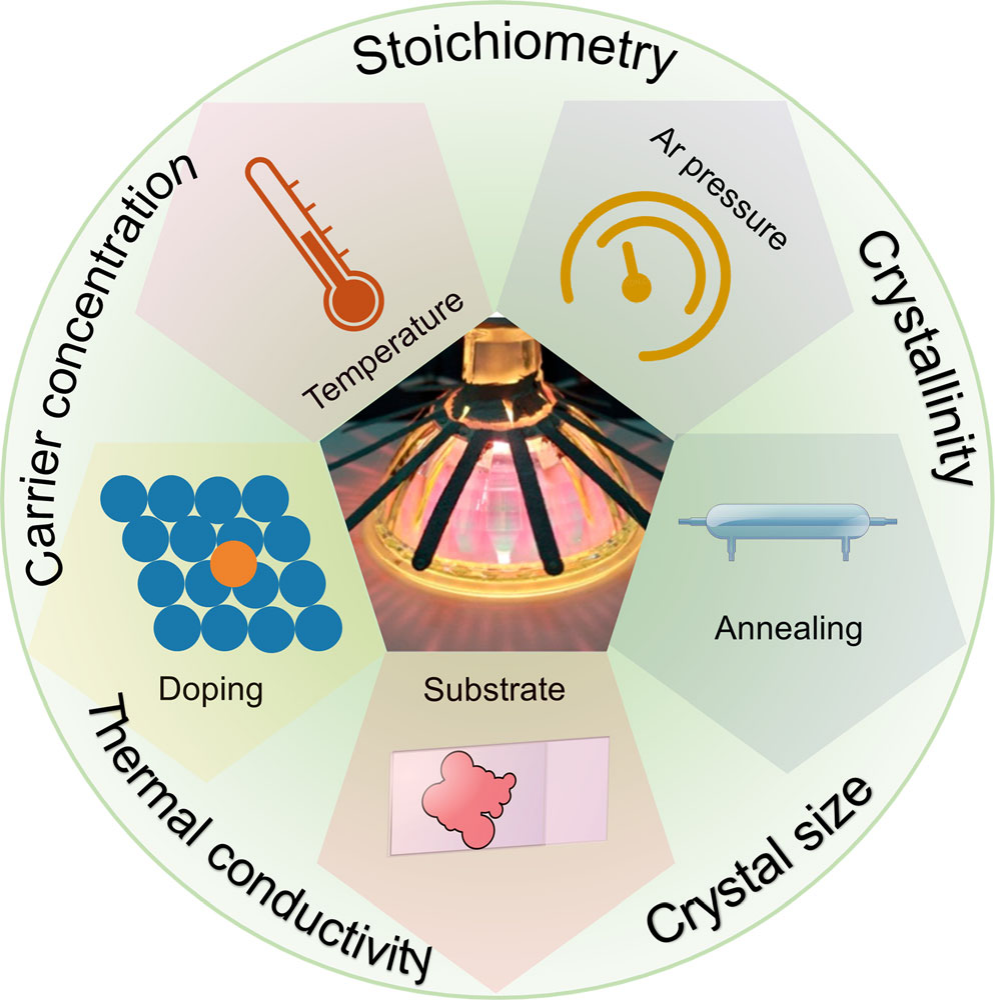
Relationship between conditions of magnetron sputtering and the performance of inorganic thermoelectric films. Center panel: Reproduced with permission.^[^ ^58^ ^]^ Copyright 2018, American Chemical Society.

Thermoelectric materials and devices show great potential in various waste heat recovery applications based on their ability in direct conversion between heat and electricity. With the development of the IoT and personal health management, an increasing demand is for smart wearable electronics, which provide a great opportunity for high‐performing thermoelectric films and their devices. Compared with other thin‐film preparation methods, magnetron sputtering is one of the best methods because of the advantages of controllable film composition, the ability to be performed at room temperature, and the possibility of using various substrates. Properties of the films can be tuned by controlling the deposition conditions (pressure, power, temperature, dopant) during the magnetron sputtering process and postprocessing after preparation. By controlling the sputtering pressure, the composition and grain orientation can be directly adjusted, and the porosity of the films can be regulated. Grain orientation and microcrystal size can be adjusted using different substrates. The substrate temperature during sputtering is a main factor to determine the chosen substrate and film flexibility. When the substrate temperature is low, organic flexible substrates can be used to maximize the flexibility of the film; while at higher sputtering temperatures, only inorganic substrates can be used. The sputtering temperature can affect the composition and the growth of the film. Besides, doping and annealing can improve the film properties, but it should be noted that if flexible organic substrates are used, the annealing time and annealing temperature need to be reduced to avoid the decomposition of the substrates that affect the film properties. In addition to the choosing of the substrate, the grain orientation and size of the prepared film are important factors in determining its flexibility. The annealing temperature and the sputtering power during magnetron sputtering affect the mechanical properties of the material. **Figure** **10** illustrates the summarized relationship between conditions of magnetron sputtering and the performance of inorganic thermoelectric films.^[^ ^58^ ^]^ As shown, the magnetron‐sputtered thermoelectric films show wide potential used in the medical field and other common waste heat recovery applications and can also be combined with photoelectric technology for applications in photosensing to supply energy to other devices.

In addition to the significant progress made in magnetron‐sputtered thermoelectric thin films and their devices, there are still considerable challenges. The *ZT* values of magnetron‐sputtered thermoelectric thin films are still lower than that of bulk materials due to the difficulty in tuning the key parameters such as *n* and *κ*_l_.^[^ ^165^, ^166^ ^]^ This is because conventional doping or alloying routes for optimizing the *n* are considerably not suitable for magnetron‐sputtered thermoelectric thin films. In addition, optimizing the grain orientation and size of the thermoelectric film is still unclear, and most of them are only experimental results, in which it is hard to guide the tuning of sputtering parameters for achieving the textured thin film. There is also less discussion on the effect of sputtering power on film properties. Besides, expanding the commercial prospects of inorganic thermoelectric films is tricky, which is mainly derived from the high cost of magnetron sputtering equipment. Expensive instrumentation makes it difficult for small‐ to medium‐sized thermoelectric manufacturers to produce high‐performance films by magnetron sputtering, thus limiting the scale of commercialization, which is further compounded by the high cost and low utilization of targets.^[^ ^167^ ^]^ The slower sputtering rate is a factor in its difficulty in commercialization. The lower sputtering rates result in low efficiency in production, making it difficult to meet market demand. Because the process utilizes a magnetic field for sputtering targets, targets with strong magnetic fields are difficult to use for magnetron sputtering, and materials such as selenides, which can easily lead to chamber poisoning, can often only be prepared by modifying the device or by combining it with other processes.^[^ ^85^, ^95^ ^]^ These limitations require further consideration of the compositions of the thermoelectric thin films and the doping elements, which complicates the optimization of the film properties. Thin films often need to be annealed to achieve high performance, and for some materials, high‐temperature annealing can lead to deformation of the flexible substrate resulting in degradation of performance and flexibility, and the way to solve the annealing of high crystallization temperature materials remains a problem. For the device, heat sink on the hot side and the degradation of performance due to long‐term wear and bending are inevitable problems. Even flexible thin‐film devices covered with PDMS still suffer from performance degradation after bending, and the mechanical properties of the devices are difficult to support practical applications that require frequent bending.

To further optimize the commercial potential of thermoelectric films made by magnetron sputtering, a few perspectives are presented here:

### 6.1 Optimization of *ZT* Values

Optimizing sputtering strategy can further increase the *ZT* values of magnetron‐sputtered thermoelectric films. By means of basic research into the material, the optimum composition ratio can be defined, and the sputtering temperature and sputtering pressure can be modulated to achieve the material the optimum composition ratio. Dopant targets can be added during the sputtering process to optimize the carrier concentration of the film. Post‐treatment with appropriate annealing and current treatment can optimize the film properties in terms of both *σ* and *κ*.^[^ ^168^ ^]^ Search new inorganic thermoelectric film material systems with high the *ZT* values are also urgent and may expand the research field.

### 6.2 Instrument Improvement

To improve the efficiency of magnetron sputtering in producing thermoelectric thin films, several factors can be considered. For one thing, proper setting of process parameters such as magnetic field strength, gas flow rate, reaction pressure, and target distance can enhance the deposition rate and uniformity of the films, thereby improving the production efficiency. For another, precise control of deposition parameters such as deposition rate and time can achieve accurate control of film thickness, composition, and structure, thus improving production efficiency and film quality. Besides, choosing targets with high purity, uniform density, high hardness, and ease of process can increase the utilization rate and durability of targets and reduce replacement frequency. Furthermore, the rotating target technology can make the target surface wear more uniform, prolong the life of the target, and at the same time increase the deposition rate and uniformity. Last, by controlling the composition and flow rate of the reactive gas, in situ reactions can be carried out during the thin‐film fabrication, thus producing films with higher quality and more complex structure and improving production efficiency.^[^ ^167^, ^169^ ^]^ However, in consideration of the organic substrates that may be used for flexible films, this strategy needs to be properly considered. To improve target utilization to save production costs, it is possible to use interpolar target hollow magnetrons, a process that can increase target utilization up to 60%.^[^ ^167^ ^]^ Alternatively, oscillating permanent magnet arrays or the creation of magnetic lines of force with two race tracks using ferromagnetic sheets can be used to increase the utilization of the target material to around 50%.^[^ ^167^ ^]^ Optimization of the tubular magnetrons invented in the early 1980s for magnetron sputtering can significantly increase the target lifetime. Although more complex than the currently used planar magnetron process, it can increase target utilization to almost 90%.^[^ ^167^ ^]^ If a magnetic material is needed for sputtering, the material needs to be a thin magnetic target to reduce the magnetic influence on the device.

### 6.3 Substrate Selection

Substrates can be used for the preparation of thermoelectric films with different applications. For devices requiring high flexibility, organic substrates can be used for sputtering, while inorganic films can be used as substrates for higher‐performance applications, ensuring the best crystal orientation of the film. Using specific types of substrates such as MgO, epitaxial films can be grown to obtain perfectly controlled crystal structures that can be used to develop the material for applications. For example, sputtering Bi_2_Te_3_ onto SWCNT allows for high performance, while using CF substrates allows for the preparation of thermoelectric films that can be cut to simplify the fabrication of devices. New types of substrates need to be investigated, allowing thermoelectric films to be prepared more easily and high‐performance devices.

### 6.4 Hybridization Preparation

For inorganic materials which are difficult to prepare by magnetron sputtering, magnetron sputtering can be combined with other conventional preparation methods. For example, Ag_2_Se can be prepared at low cost by sputtering Ag onto a substrate and immersing it in an aqueous Se/Na_2_S solution, in addition to redesigning the magnetron sputtering device.^[^ ^95^ ^]^ Using hybridization methods, a wider range of high‐quality inorganic thermoelectric films can be produced at reduced magnetron sputtering costs. For some IV cluster thermoelectric materials, high‐temperature annealing can be accomplished at lower temperatures by interlayer exchange methods to balance the flexibility as well as the performance of the film.

### 6.5 Optimization of Mechanical Properties of Thin‐Film Devices

In term of flexible wearable devices, the connection of the device electrodes and the bending ability of the thermoelectric film need to be considered and optimized. An integrated circuit can be used, and the complete circuit can be etched on the flexible circuit board to reduce the electrodes falling off when the device is bent. The flexible inorganic thermoelectric film prepared by magnetron sputtering needs to be tightly wrapped by flexible material to reduce its degradation after bending, and the use of flexible substrates with special surface structures makes the film more flexible and reduce the degradation of performance due to bending.

## Conflict of Interest

The authors declare no conflict of interest.
